# Inhibition of the inflammatory response to stress by targeting interaction between PKR and its cellular activator PACT

**DOI:** 10.1038/s41598-017-16089-8

**Published:** 2017-11-23

**Authors:** Stephanie Dabo, Patrick Maillard, Milagros Collados Rodriguez, Marianne Doré Hansen, Sabrina Mazouz, Donna-Joe Bigot, Marion Tible, Geneviève Janvier, Olivier Helynck, Patricia Cassonnet, Yves Jacob, Jacques Bellalou, Anne Gatignol, Rekha C. Patel, Jacques Hugon, Hélène Munier-Lehmann, Eliane F. Meurs

**Affiliations:** 1 Unité Hepacivirus and Innate Immunity, Institut Pasteur, 75015 Paris, France; 2 CNRS, UMR 3569 Paris, France; 3 Department of Clinical and Molecular Medicine, Faculty of Medicine and Health Sciences, Norwegian University of Science and Technology, 7006 Trondheim, Norway; 4 Center of Cognitive Neurology, Lariboisière Hospital AP-HP University Paris Diderot, Paris, France; 5 Inserm, U942 Paris, France; 6 Unité de Chimie et Biocatalyse, Institut Pasteur, 75015 Paris, France; 7 CNRS, UMR3523 Paris, France; 8 Unité de Génétique Moléculaire des Virus à ARN, Institut Pasteur, Université Paris Diderot, Paris, France; 9 Plate-forme des protéines recombinantes, Institut Pasteur, 75015, CNRS UMR 3528 Paris, France; 10 Virus-Cell Interactions Laboratory, Lady Davis Institute for Medical Research, Department of Medicine, department of Microbiology and Immunology, McGill University, Montreal, Quebec Canada; 11 University of South Carolina, Department of Biological Sciences, Columbia, South Carolina 29208 USA

## Abstract

PKR is a cellular kinase involved in the regulation of the integrative stress response (ISR) and pro-inflammatory pathways. Two N-terminal dsRNA Binding Domains (DRBD) are required for activation of PKR, by interaction with either dsRNA or PACT, another cellular DRBD-containing protein. A role for PKR and PACT in inflammatory processes linked to neurodegenerative diseases has been proposed and raised interest for pharmacological PKR inhibitors. However, the role of PKR in inflammation is subject to controversy. We identified the flavonoid luteolin as an inhibitor of the PKR/PACT interaction at the level of their DRBDs using high-throughput screening of chemical libraries by homogeneous time-resolved fluorescence. This was further validated using NanoLuc-Based Protein Complementation Assay. Luteolin inhibits PKR phosphorylation, the ISR and the induction of pro-inflammatory cytokines in human THP1 macrophages submitted to oxidative stress and toll-like receptor (TLR) agonist. Similarly, luteolin inhibits induction of pro-inflammatory cytokines in murine microglial macrophages. In contrast, luteolin increased activation of the inflammasome, in a PKR-independent manner. Collectively, these data delineate the importance of PKR in the inflammation process to the ISR and induction of pro-inflammatory cytokines. Pharmacological inhibitors of PKR should be used in combination with drugs targeting directly the inflammasome.

## Introduction

PKR (Protein Kinase dsRNA-dependent) is one of the four eIF2α kinases which controls general protein translation and concomitantly triggers the integrative stress response through the eIF2α-independent enhanced translation of transcription factors such as ATF4^1^. In addition, PKR participates in the NF-κB signaling pathways leading to induction of pro-inflammatory cytokines. For this activation, PKR may act through its kinase activity or also through protein/protein interaction^2–8^. A link between PKR and the inflammasome was also reported but here, the situation is less clear as PKR has been proposed to participate in the assembly of the inflammasome, dependent^4^ or not of its kinase activity^6^, to have no effect^8^ or to diminish inflammasome activity through its control on translation^5^. Understanding the role of PKR in the inflammation process is of particular interest in view of studies indicating its participation in neurodegenerative diseases and other human pathologies related to inflammation. For instance, following a study showing that phosphorylation of eIF-2α was impairing memory formation^9^, cognitive studies with PKR deficient mice revealed that suppression of PKR promotes network excitability and enhanced cognition^10^.

The N-terminus of PKR contains two basic helical domains referred to as dsRNA Binding Domains (DRBD) through which PKR binds to dsRNA or to other DRBD-containing proteins. One of these, the cellular PACT protein (PKR Activator) interacts with PKR in response to a variety of cellular stresses, such as those resulting from perturbations of the endoplasmic reticulum or the oxidative phosphorylation function of the mitochondria. PACT has been demonstrated to activate PKR *in vitro* as well as *in vivo* after induction by an oxidative stress^11–16^. Indeed, such a stress prevents PACT to be sequestered as an inactive heterodimer with the TAR RNA Binding Protein (TRBP) and releases its PKR activation ability^17,18^. Colocalisation of PACT with phosphorylated PKR was observed by immunohistochemistry in the cytoplasm of hippocampal neurons of post-mortem brains of patients whith Alzheimer’s disease, in line with a possible role for PKR in cognitive disorders^19^. Furthermore, oxidative stress can increase, in a PKR-dependent manner, the translation of BACE1 (beta-site APP cleaving enzyme 1), the rate-limiting enzyme involved in the generation of amyloid β (Aβ)-peptide^20^. In the brain, Aβ is known to bind to the microglial receptor complex CD36/TLR4-6 and trigger induction of pro-inflammatory cytokines, such as IL-8, IL-6 and IL1-β, similar to the action of microbial effectors, such as LPS^21^. While IL-8 and IL6 are directly released from the cells under their active form, production of IL1-β requires activation of the inflammasome for its cleavage by caspase-1 from the pro-IL1-β form. Formation of the NLRP3 inflammasome complex^22^ can occur following Aβ phagocytosis and subsequent lysosomal damage which activates an oxidative stress through the plasma membrane-localized NADPH oxidase (Nox2)^23,24^. It is possible that PKR could be involved both in the generation of Aβ through its eIF-2α kinase activity and in the action of Aβ through NF-κB signaling and regulation of the inflammasome, thus raising interest to generate PKR inhibitors in order to be able to deal with neurodegenerative pathologies.

A limited number of PKR inhibitors have been previously described. Screening 26 different ATP-binding site inhibitors to target the catalytic activity of PKR led to the isolation of the oxindole/imidazole derivative C16^25^. Inhibiting PKR activation at the level of its N-terminus was demonstrated by using a cell penetrating peptide, referred to as PRI, which contains the 21-aa peptide corresponding to the first DRBD of PKR^26^. A different approach by high-throughput screening aimed at identifying molecules that protect macrophages from anthrax lethal toxin-induced cell death through NLRP1 inflammasome activation, led to the identification of a compound (7-desacetoxy-6,7-dehydrogedunin (7DG)) that binds to the C-terminus of PKR but does not interfere with the PKR kinase activity^6^.

Here, we have performed a high-throughput screening of chemical libraries to isolate molecules that can interfere with the interaction of the N-terminus of PKR with its cellular activator PACT, in order to identify novel inhibitors of PKR and to better understand the mechanism of action of PKR on the signaling pathways linked to inflammation.

## Results

### Set up of *in vitro* PKR/PACT interaction by HTRF and screening of libraries of chemical compounds

**Figure 1:**
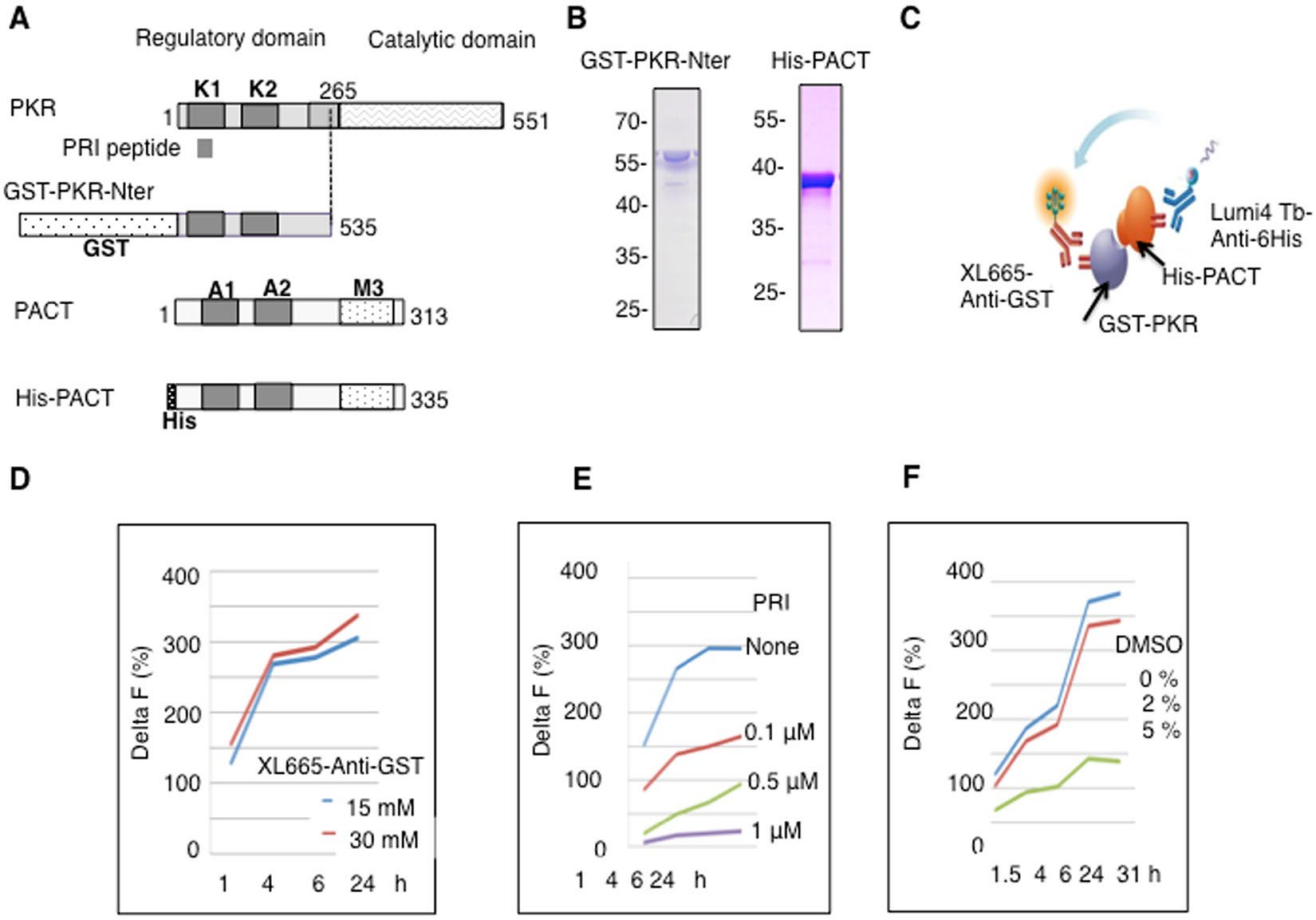
*In vitro* interaction of PKR and PACT by HTRF. (**A**) Schematic representation of full length PKR, PKR 1–265 fragment in fusion with GST at its N-terminus, PACT and PACT in fusion with His tag at its N-terminus. The position of the two Double Stranded RNA Binding Domains are indicated (respectively K1 and K2 for PKR and A1 and A2 for PACT) as well as the M3 domain of PACT. The PKR-Nter fragment ends at aa 265, located in the 234–275 basic domain of PKR (grey box). PRI peptide corresponds to the amino-acids 60 to 80 of first DRBD of PKR. (**B**) Analysis of the GST-PKR-Nter and His-PACT proteins by SDS-PAGE after purification on glutathione-sepharose and on Ni-charged His-bind resin, respectively. Gel analysis shows the profile of GST-PKR-Nter and His-PACT after purification. The purified fractions were pooled and processed for resuspension as soluble proteins as described in Materials and Methods. (**C**) schematic representation of the HTRF assay (Cisbio) showing transfer of energy from the donor fluorophore (Lumi4Tb) to the acceptor (XL665), each coupled to anti-6His or anti-GST antibodies, when GST-PKR and His-PACT are interacting. (**D**) GST-PKR and His-PACT (100 nM each) were mixed and incubated for 30 min at 30 °C before addition of 4 nM of anti-6His Lumi4Tb antibodies and either 15 or 30 nM of anti-GST XL665 antibodies. (**E**) GST-PKR (100 nM) was first incubated for 30 min with different concentrations of PRI as indicated before addition of His-PACT (100 nM). After another 30 min of incubation, the anti-6His Lumi4Tb (4 nM) and anti-GST XL665 (15 nM) antibodies were added. (**F**) GST-PKR and His-PACT (100 nM each) were mixed and incubated for 30 min at 30 °C before addition of DMSO at the indicated concentrations. After another 30 min, the anti-6His Lumi4Tb (4 nM) and anti-GST XL665 (15 nM) antibodies were added. Incubation and reading of fluorescence were performed for the indicated times. The percentage of Delta F was calculated as described in Materials and Methods.

We set up an *in vitro* Homogeneous Time-Resolved Fluorescence (HTRF) interaction assay between PKR and PACT, using purified preparations of His-PACT and GST-PKR-Nter (Fig. 1A,B). The two proteins were incubated in the presence of the acceptor XL665 –labeled anti-GST antibody which binds to the GST moiety of the PKR construct and the donor Lumi4 Tb labeled anti-6His antibody, which binds to the His tag of His-PACT (Fig. 1C). Interaction between the two proteins resulted in transfer of energy and fluorescence which can be expressed as percentage of ΔF (see Materials and Methods). Maximum fluorescence signal was obtained after 6 hrs and did not vary over 24 hrs. Experiments reproducibly showed a ΔF value around 300–400 (Fig. 1D). Addition of an excess of the PRI peptide corresponding to the sequence located in the first DRBD of PKR^26^ strongly inhibited fluorescence, thus validating our assay (Fig. 1E). DMSO, at concentration up to 2% did not significantly affect the interaction (Fig. 1F), allowing to conveniently dilute the chemical compounds at a final concentration of 1% DMSO for the screening procedure. The PKR/PACT HTRF interaction assay was then used to screen the Prestwick Chemical Library® (1,200 compounds with high chemical and pharmacological diversity) and a total collection of 38,173 compounds, coming from the ≪ Chimiothèque Nationale ≫ and CHEM-X-INFINITY (see Materials and Methods). The screening procedure yielded 113 hits that could inhibit the transfer of fluorescence higher than 95%. Two successive HTRF assays were then performed on these hits to determine the specificity of the inhibition as well as their IC50 for efficiency of inhibition and for cytotoxicity (Supplementary Table 2) This resulted in the selection of 2 compounds from the Prestwick library (myricetin and quercetin) and 14 compounds from the other libraries, among which luteolin, a natural polyphenol, member of the flavonoid family and similar to myricetin and quercetin. Interestingly, a number of studies have reported anti-inflammatory effects for these flavonoids and interest in therapy^27^. The next step was to evaluate the effect of the selected compounds on the PKR/PACT interaction in a cellular context.

### Flavonoids inhibit the PKR/PACT interaction at the level of their DRBDs

**Figure 2:**
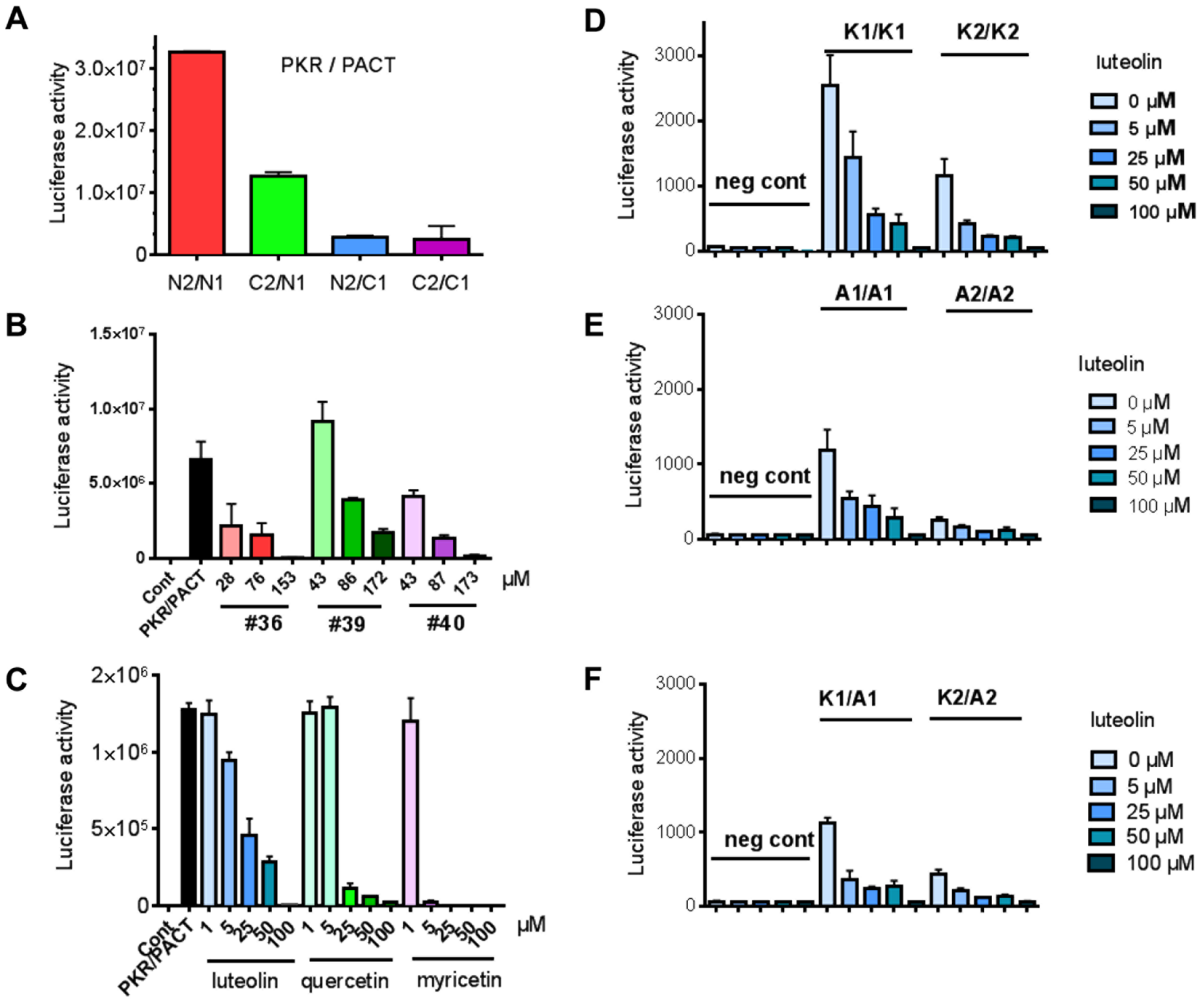
Effect of compounds on the PKR/PACT interaction monitored by NPCA. (**A**) HEK 293 T cells (30,000 cells per well in 96-well plates) were transfected with 100 ng of PKR-Nter or PACT plasmids bearing the Nanoluc moiety 1 or 2 at their N- or C-terminus, as indicated. At 24 h post-transfection, the medium of the cells was discarded and the cells were incubated in the presence of 50 μl of Nano-Glo reagent. Luciferase enzymatic activity was measured using a Berthold Centro XS LB960 luminometer and counting luminescence for 5 s. The values represent the mean of triplicate experiments. Saturation of luciferase signal was found to occur frequently in the HEK293T cells and the subsequent NPCA experiments were then performed in the Huh7.5/CD81 cells, which proved to give less intense and still satisfactory luciferase signal. (**B**,**C**) Huh7.25/CD81 cells in 96-well plates were transfected with 100 ng each of PKR-N2 and PACT-N1. They were then either untreated or incubated with different concentrations of the Lyon compounds (referred to as #36, #39 and #40) (**B**), or luteolin, quercetin and myricetin (**C**) as indicated. Treatment was done either at the time of transfection or up to 6 hrs post transfection with identical effect on the luciferase activity. Luciferase enzymatic activity was measured after 24 hrs. The values represent the mean of triplicate experiments. (**D**,**F**) The DRBDs of PKR or PACT were cloned in fusion with the moeity 1 or 2 of luciferase at their N-terminus. The Huh7.25/CD81 cells in 96-well plates were transfected with 100 ng of the resulting plasmids expressing the first or second DRBD of PKR (K1 or K2; (**D**), or PACT (A1 or A2; (**E**) in homodimerization (**D**,**E**) or heterodimerization (**F**) configuration. The cells were either untreated or incubated with different concentrations of luteolin. Luciferase enzymatic activity was measured after 24 hrs. The values represent the mean of triplicate experiments.

Based on a previous experience with a split-luciferase protein-protein interaction detection system^28^, we designed a split-Nanoluc luciferase complementation assay (NPCA) to examine the effect of the compounds on the interaction between PKR and PACT in a cellular context, using HEK-293T cells. For the NPCA assay, we constructed plasmids expressing the N-terminus of PKR (1–265 aa) or the full length PACT with the two complementary nanoluc moieties (referred to as 1 and 2) inserted in all available combinations at the N- or C-terminus of PKR or PACT^29^. The highest efficiency of interaction, revealed after cotransfection of the different constructs, was obtained when these moieties are placed at the N-terminus of PKR and PACT, giving the combination PKR-N2/PACT-N1 (Fig. 2A). The cells were then cotransfected with this pair of plasmids and 4 hrs later, incubated in the presence of different concentrations of the compounds. Luciferase activity was measured after 24 hrs. The results are shown for the compounds that could significantly inhibit luciferase activity (Fig. 2B,C). To determine whether the inhibition of the PKR/PACT interaction occurred at the level of their DRBDs (referred to as K1, K2 for PKR and A1, A2 for PACT; see Fig. 1A), each of the DRBDs was cloned in fusion with the luciferase moieties at their N-terminus and submitted to a second NPCA. The results first confirmed the ability of the DRBDs to homodimerize, whether for PKR (K1/K1 or K2/K2; Fig. 2D) or for PACT (A1/A1 or A2/A2; Fig. 2E) as well as to heterodimerize (K1/A1 and K2/A2; Fig. 2F) as shown previously by yeast two hybrid assay^30^. Importantly, we found that interaction between the DRBDs, whether in the homo-or hetero-dimerization configuration, was efficiently inhibited by luteolin at 5 μM (Fig. 2D,F). Of note, this experiment was done only for luteolin since the other compounds did not inhibit PKR phosphorylation effectively enough in the subsequent assays and were therefore discarded. Altogether, these data reveal for the first time that the PKR/PACT association can be specifically disrupted by members of the flavonoid family and that this occurs at the level of their DRBDs.

### Luteolin strongly inhibits PKR phosphorylation in a cellular stress assay

**Figure 3:**
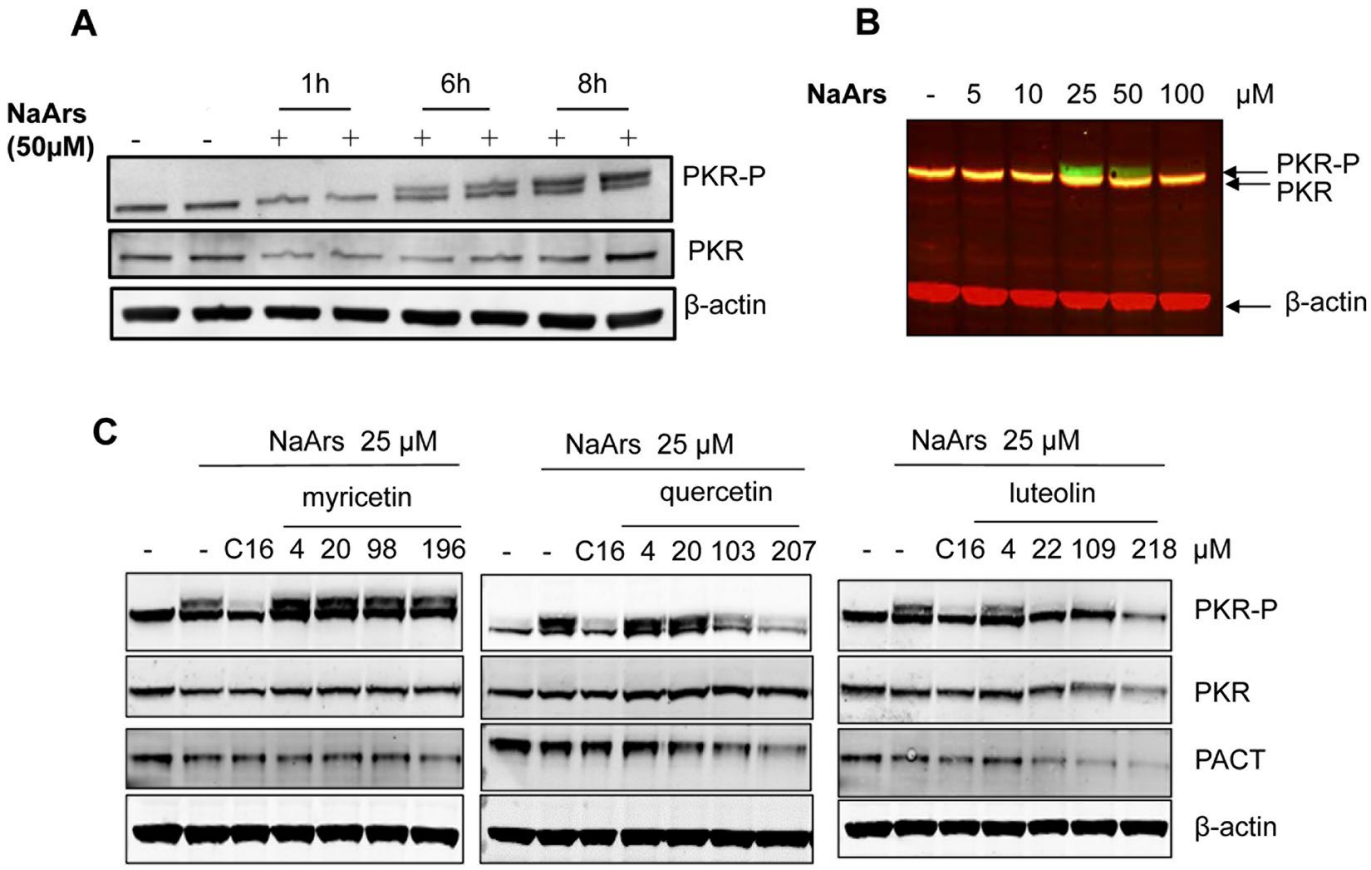
Inhibition of PKR phosphorylation by luteolin in THPI macrophages submitted to stress. The THP1 cells were differentiated by PMA treatment for 72 hrs as described in Materials and Methods. (**A**) The cells were incubated for the indicated times in the absence or presence of 50 μM sodium arsenite (NaArs). (**B**) The cells were incubated for 8 hrs in the presence of different concentrations of NaArs as indicated. The relative migration of total levels of PKR (red) and phosphorylated PKR (green) is shown. (**C**) The THP1 cells were either untreated or incubated for 30 min with C16 (400 nM) or different concentrations of myricetin, quercetin or luteolin, after which they were incubated in the presence of 25 μM NaArs. Lysates were prepared and proteins were separated by SDS-PAGE prior to fluorescent immunoblot analysis (Odyssey Imaging system; Li-Cor). Detection of β-Actin served as control.

We then used the human THP1 macrophages as a convenient cellular model for subsequent analysis of the effects of the compounds on PKR activation as well as on induction of pro-inflammatory cytokines and activation of inflammasome. PKR activation was correctly detected upon treatment of the cells with sodium arsenite, inducing oxidative stress known to trigger PKR activation through PACT in accord with previous reports^15,31^. Kinetics experiment showed an increase in PKR phosphorylation with the maximum effect at 8 hrs post treatment (Fig. 3A) and a dose response experiment showed maximum phosphorylation for 25 μM of sodium arsenite (Fig. 3B).The cells were then incubated in the presence of luteolin, myricetin and quercetin for 30 min before incubation with 25 μM of sodium arsenite for 8 hr, and the cell extracts were analysed for PKR activation. Treatment of cells with 400 nM of C16, a small molecule inhibitor of the catalytic activity of PKR^25^, was included in the assay as control and the degree of efficiency of the compounds to inhibit PKR was estimated by comparison with the action of C16 (Fig. 3C). Luteolin presented the best ability to inhibit phosphorylation of PKR, with inhibition starting at 4 µM and being maximal around 20 µM. A ten-fold higher concentration (200 µM) was required for quercetin, while myricetin had no effect (Fig. 3C). Some decrease in the expression levels of PACT and PKR was observed for the highest concentrations of luteolin. This may indicate their enhanced susceptibility to degradation in response to stress when they are not longer in association. Altogether, these data show that, out of the three flavonoids that were identified as disrupting the PKR/PACT interaction by both HTRF and NPCA, luteolin was the most efficient as a specific inhibitor of PKR phosphorylation in response to oxidative stress.

### Activation of the integrative stress response (ISR) and induction of pro-inflammatory cytokines in THP1 cells upon treatment with sodium arsenite or thapsigargin and with an LPS analog

**Figure 4:**
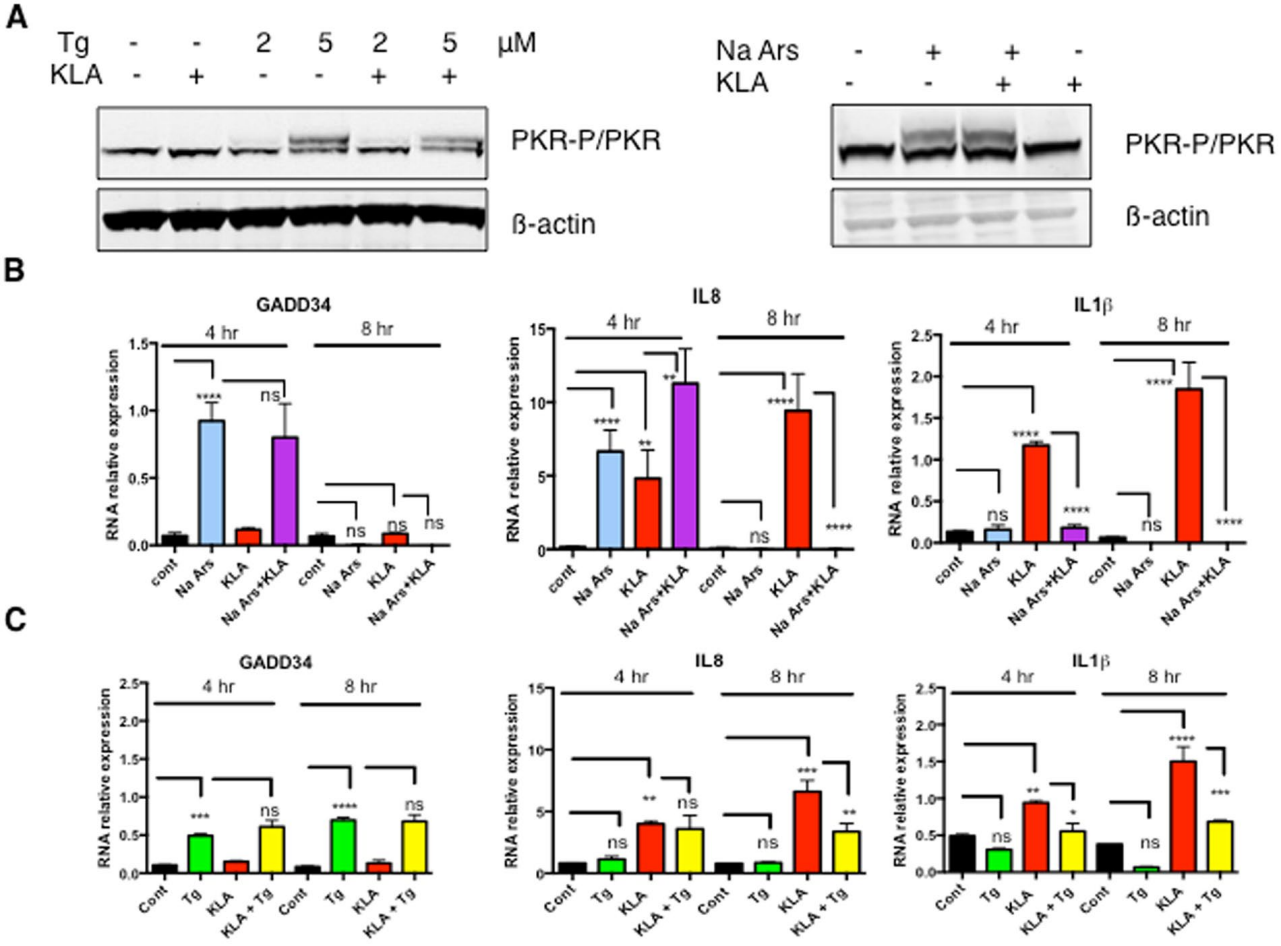
Induction of integrative stress response and pro-inflammatory cytokines in THP1. The THP1 cells were differentiated by PMA treatment for 72 hrs as described in Materials and Methods. (**A**) The THP1 cells were either untreated or incubated for 8 hrs in the presence of 2 or 5 μM of thapsigargin (Tg), KLA (100 ng/ml) or both drugs (left), or in the presence of 25 μM sodium arsenate (NaArs), KLA (100 ng/ml) or both drugs (right). Lysates were prepared and proteins were separated by SDS-PAGE prior to fluorescent immunoblot analysis of PKR and phosphorylated PKR. Detection of β-Actin served as control. (**B**,**C**) The cells were incubated for the indicated times in the absence or presence of 25 μM sodium arsenite (NaArs), 100 ng/ml KLA, either separated or together (**B**) or in the absence or presence of 5 μM of thapsigargin (Tg), 100 ng/ml KLA, either separated or together (**C**). Total RNA was isolated and the levels of GADD34, IL-8 and IL-1ß were determined by RT-qPCR. Data are expressed as RNA relative expression after normalization with GAPDH. Data represents means ± SD from three experiments; *P < 0.05, **P < 0.005, ***P < 0.0005, ****P < 0.00005, ns: non significant, Tukey’s multiple comparison test in ANOVA.

In the next assay, we submitted THP1 cells to oxidative stress to trigger activation of PKR as a kinase and to treatment with a TLR agonist to activate the NF-κB signaling pathway, in which PKR can participate as a scaffolding partner^2^. This allowed to examine the effect of luteolin on these pathways. Oxidative stress was triggered upon treatment with either sodium arsenite or thapsigargin, another inducer of oxidative stress known to trigger PKR activation through PACT^31^. As TLR agonist, we used Kdo_2_-Lipid A (KLA), a defined LPS from *E*. *coli* which triggers NF-κB activation through TLR4^32^. Induction of the ATF4-dependent GADD34 gene and of the pro-inflammatory cytokines IL-8 and pro-IL-1β were chosen as markers of the integrative response to stress and NF-κB activation, respectively. We first showed that similar to sodium arsenite, thapsigargin triggers PKR phosphorylation. In contrast, KLA did not trigger PKR phosphorylation, as expected since activation of NF-κB does not require the kinase function of PKR, and did not interfere with the effect of thapsigargin and sodium arsenite on this phosphorylation (Fig. 4A). THP1 macrophages were then treated for 4 and 8 hrs with either sodium arsenite (Fig. 4B) or thapsigargin (Fig. 4C). The results showed that GADD34 was induced by sodium arsenite or thapsigargin with a more sustained induction over time by thapsigargin treatment and that it was not significantly affected by KLA treatment (Fig. 4B,C; left). Conversely, induction of the NF-κB-dependent genes IL8 and pro-IL1β was responding almost exclusively to KLA (Fig. 4B,C; middle and right). In addition, we observed that Na Arsenate or thapsigargin treatment interfered with induction of pro-inflammatory cytokines by KLA. Oxidative stress is also known to activate the Nrf2 pathway, independently of PKR or PACT and this has been shown to occur in response to arsenic^33^ or thapsigargin^34^. Therefore, it is possible that this observed inhibition might be the result of the well known cross-talk between the NF-κB and Nrf2 response pathways through competition at the transcriptional level, for binding to the transcriptional co-activator CBP (CREB-binding)-p300 complex^35^. However, thapsigargin proved to be less inhibitory that Na Arsenate and thapsigargin/KLA was therefore chosen over sodium arsenite/KLA to be able to correctly evaluate the effect of the PKR inhibitor on the following events of the inflammatory response in which PKR is thought to participate: the integrative stress response, the NF-κB activation pathway and the inflammasome.

### Luteolin inhibits the integrative stress response and induction of pro-inflammatory cytokines in THP1 and in murine primary macrophages

**Figure 5:**
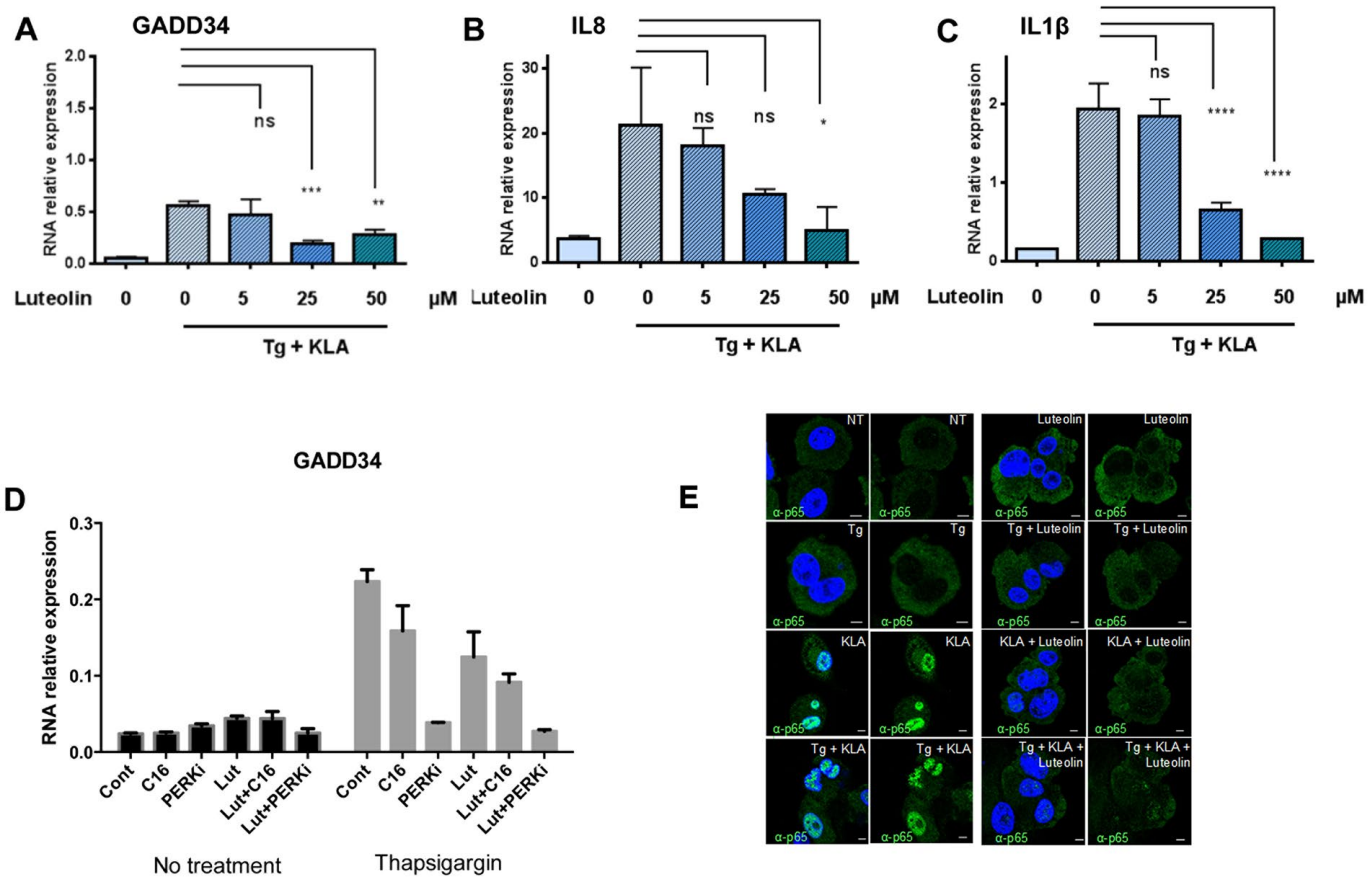
Luteolin inhibits the integrative stress response and induction of pro-inflammatory cytokines in THP1. The THP1 cells were differentiated by PMA treatment for 72 hrs as described in Materials and Methods. (**A**,**C**) The cells were either untreated (0) or treated with 5, 25 or 50 μM of luteolin. After 30 min, the cells were incubated in the presence of 5 μM of thapsigargin and 100 ng/ml KLA for 8 hrs. (**D**) The cells were either untreated (Cont) or treated with for 30 min with C16 (400 nM), PERKi (2 μM), or luteolin at a concentration of 25 μM, either alone (Lut) or in combination with C16 (Lut + C16) or PERKi (Lut + PERKi). The cells were then incubated in the presence of 5 μM of thapsigargin for 8 hrs. Total RNA was isolated and the levels of GADD34 (**A** or **D**), IL-8 (**B**) and IL-1ß (**C**) were determined by RT-qPCR. Data are expressed as RNA relative expression after normalization with GAPDH. Data represents means ± SD from three wells; *P < 0.05, **P < 0.005, ***P < 0.0005, ****P < 0.00005, ns: non significant, Tukey’s multiple comparison test in ANOVA. (**E**) the cells were either untreated (NT) or treated with 50 μM of luteolin. After 30 min, the cells were incubated in the presence of 5 μM of thapsigargin (Tg), 100 ng/ml KLA or both (Tg + KLA) for 60 min. Cells were fixed with 4% PFA, blocked and immunostained with anti-NFκB p65 polyclonal antibodies (green). Nuclei were visualized with DAPI (blue).

We then submitted the THP1 cells to the combined treatment thapsigargin/KLA in the presence of different concentrations of luteolin. The results showed that luteolin inhibited induction of GADD34, IL8 and pro-IL1β (Fig. 5A–C). We noticed a residual induction of GADD34, even for the highest concentration of luteolin used. This residual induction could result from the activation of PERK, another eIF2α kinase, which can also be activated by thapsigargin, through Endoplasmic Reticulum (ER) stress^36^. To address this, we compared the effect of luteolin to that of inhibitors of the catalytic activity of PKR (C16) or PERK (PERKi). The effect of luteolin was found to be similar to that of C16 and both were less effective than the PERK inhibitor (Fig. 5D). These data show that, in conditions where PKR and PERK are both activated to trigger the integrative stress response, treatment with luteolin is able to significantly attenuate this effect, but not to inhibit it completely.

Because of the possible interference by the Nrf2/ NF-kB cross-talk in our assay, it was possible that the observed reduced induction of pro-inflammatory cytokines by KLA in the presence of thapsigargin might mask a role of the PKR/PACT association in the early events of NF-kB activation. To examine this, we analysed NF-kB activation 60 min post-treatment with the drugs through translocation of NF-kB p65 protein in the nucleus by confocal microscopy. The results show that only KLA triggers the translocation of NF-kB, whether alone or in the presence of thapsigargin and that luteolin can inhibit this translocation. Therefore, the ability of luteolin to attenuate the induction of the pro-inflammatory cytokines is independent from its effect on the PKR/PACT association (Fig. 5E).

**Figure 6:**
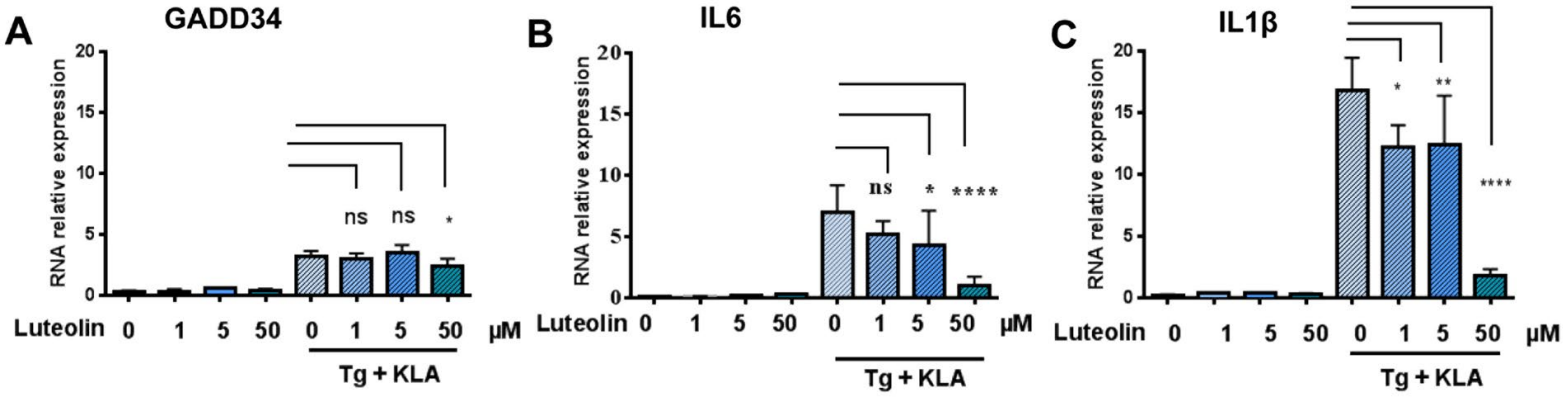
Luteolin inhibits the integrative stress response and induction of pro-inflammatory cytokines in murine primary macrophages. Microglia from hippocampal tissues of C57/BL6 embryos was prepared as described in Materials and Methods. The cells, in Poly L-ornithine-coated dishes, were incubated in the presence of 1, 5 or 50 μM of luteolin for 30 min and either treated or not with 5 μM of thapsigargin and 100 ng/ml KLA for 4 hrs. Supernatants were collected for ELISA analysis of cytokines. Total RNA was isolated from harvested cells and levels of GADD34 (**A**), IL-6 (**B**) and IL-1ß (**C**) were determined by RT-qPCR. Data are expressed as RNA relative expression. Data represents means ± SD from three independent dishes; *P < 0.05, **P < 0.005, ****P < 0.00005, ns: non significant, Tukey’s multiple comparison test in ANOVA.

The ability of luteolin to inhibit induction of pro-inflammatory cytokines was then assayed on primary macrophages, using microglia isolated from the hippocampal regions of murine embryos. In this experiment, the microglial cells were submitted to 4 hrs of treatment with 5 μM thapsigargin and 100 ng/ml KLA, in the absence or presence of different concentrations of luteolin (1, 5 and 50 μM). The murine microglial cells proved to respond well to the stress situation by induction of significant induction of the murine pro-inflammatory markers IL6 (6-fold) or IL1β (15-fold). Luteolin was found to inhibit these inductions in a concentration manner, with maximum effect for 50 µM and an already significant effect at 5 µM (Fig. 6). We noted that the oxidative stress response (induction of GADD34) was not as sensitive to luteolin as induction of pro-inflammatory cytokines; only a slight but significant inhibition was detected at 50 μM. Altogether, these data show that luteolin can interfere with the cellular response to stress by limiting the induction of pro-inflammatory cytokines in cells of two different mammalian species.

### The PKR inhibitor luteolin increases the activation of inflammasome

**Figure 7:**
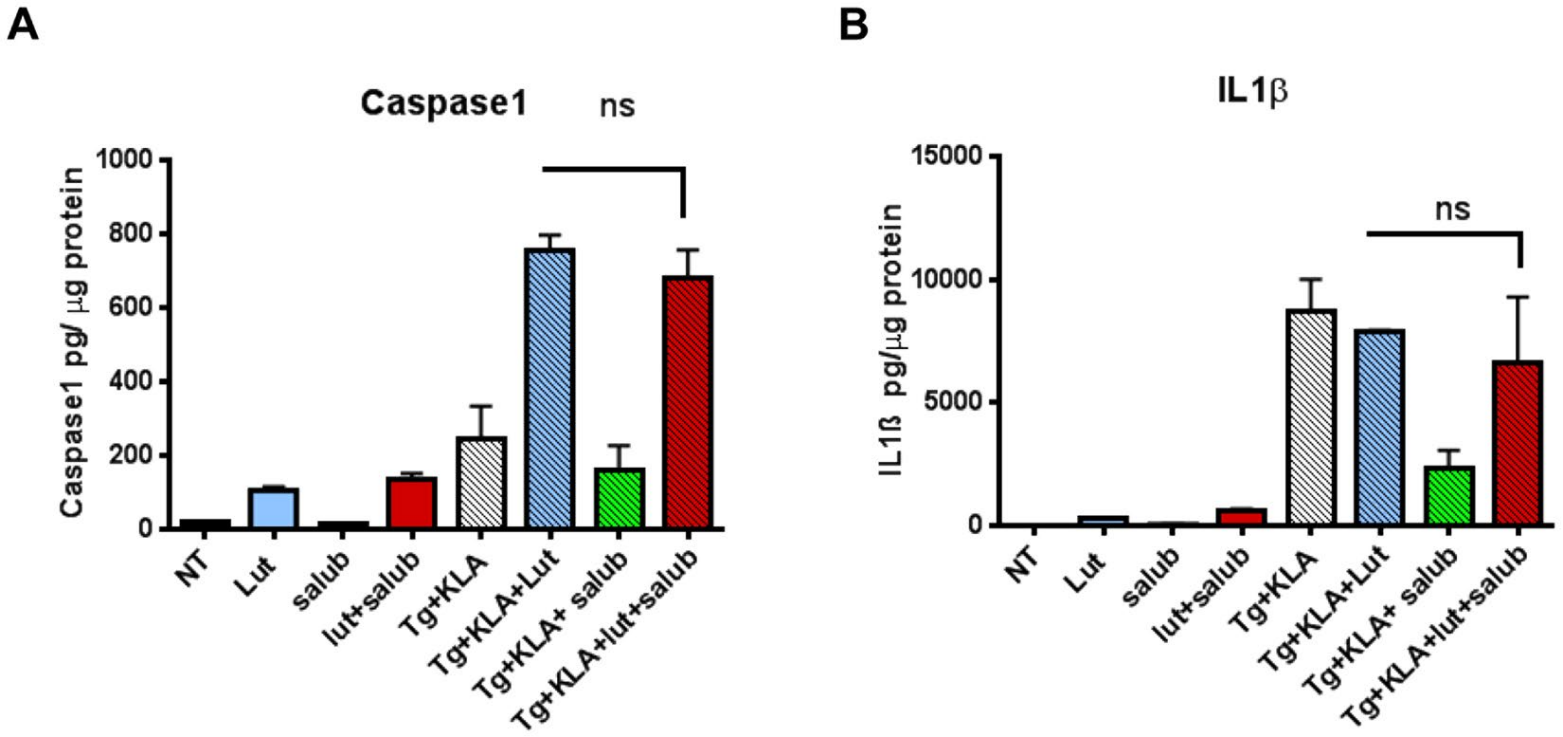
The PKR/PACT inhibitor Luteolin triggers activation of the inflammasome. The THP1 cells were differentiated by PMA treatment for 72 hrs as described in Materials and Methods. The cells were either untreated (NT) or treated with 50 μM of luteolin (Lut) or 10 μM of Salubrinal (salub) alone. After 30 min, incubation was continued for 8 hrs as such or in the presence of 5 μM of thapsigargin and 100 ng/ml KLA (Tg + KLA). Where indicated, the cells were treated with luteolin in the presence of each of the other drugs. After 8 hrs, activation of Caspase-1 (**A**) and secretion of IL-1ß (**B**) in the supernatants were determined by ELISA and the values were normalised to the amount of total protein for each condition. Data represents means ± SD from three independent supernatants.

We then examined the effect of luteolin on the activation of NLRP3 inflammasome. Such activation can take place following an increase in the NLRP3 expression through NF-κB (priming event) and its assembly as a complex. The latter can be triggered in response to several different stimuli, in particular via oxidative stress. Once activated, the inflammasome triggers activation of caspase-1, subsequent cleavage of pro-IL-1β and secretion of the resulting IL-1β as well as secretion of caspase-1. Activation of NF-κB by KLA and activation of oxidative stress by thapsigargin can here be conveniently used to study activation of the inflammasome and, accordingly, we showed that treatment of THP1 cells with thapsigargin/ KLA could trigger the expression of caspase-1 and IL-1β as measured by ELISA (Fig. 7). We found that treatment with luteolin further increased the expression of Caspase-1 (Fig. 7A). These data could agree with a previous report^5^ showing that PKR negatively controls the inflammasome. By preventing PKR to negatively control translation, luteolin would then favour the translation of effectors required for the formation of the inflammasome complex and subsequently its activation. Although no increase in the expression of IL-1β was observed after luteolin treatment (Fig. 7B), this could be explained because of the ability of luteolin to first inhibit its induction through NF-κB (see Fig. 5). To determine whether the hypothesis that PKR controls the inflammasome through translation was correct, we compared the action of luteolin to that of Salubrinal^37^ which can strongly inhibit translation by preventing the dephosphorylation of eIF2α and therefore acts downstream of PKR. If luteolin restores translation by inhibiting PKR, then its positive effect would be lost in the presence of salubrinal. The results showed that, on the contrary, luteolin kept its ability to increase the expression of Caspase-1 or IL-1β in the presence of Salubrinal (Fig. 7A,B). Therefore, these data are arguing against a role for PKR in controlling the inflammasome through translation.

### PKR and PACT are not involved in the activation of the inflammasome

**Figure 8:**
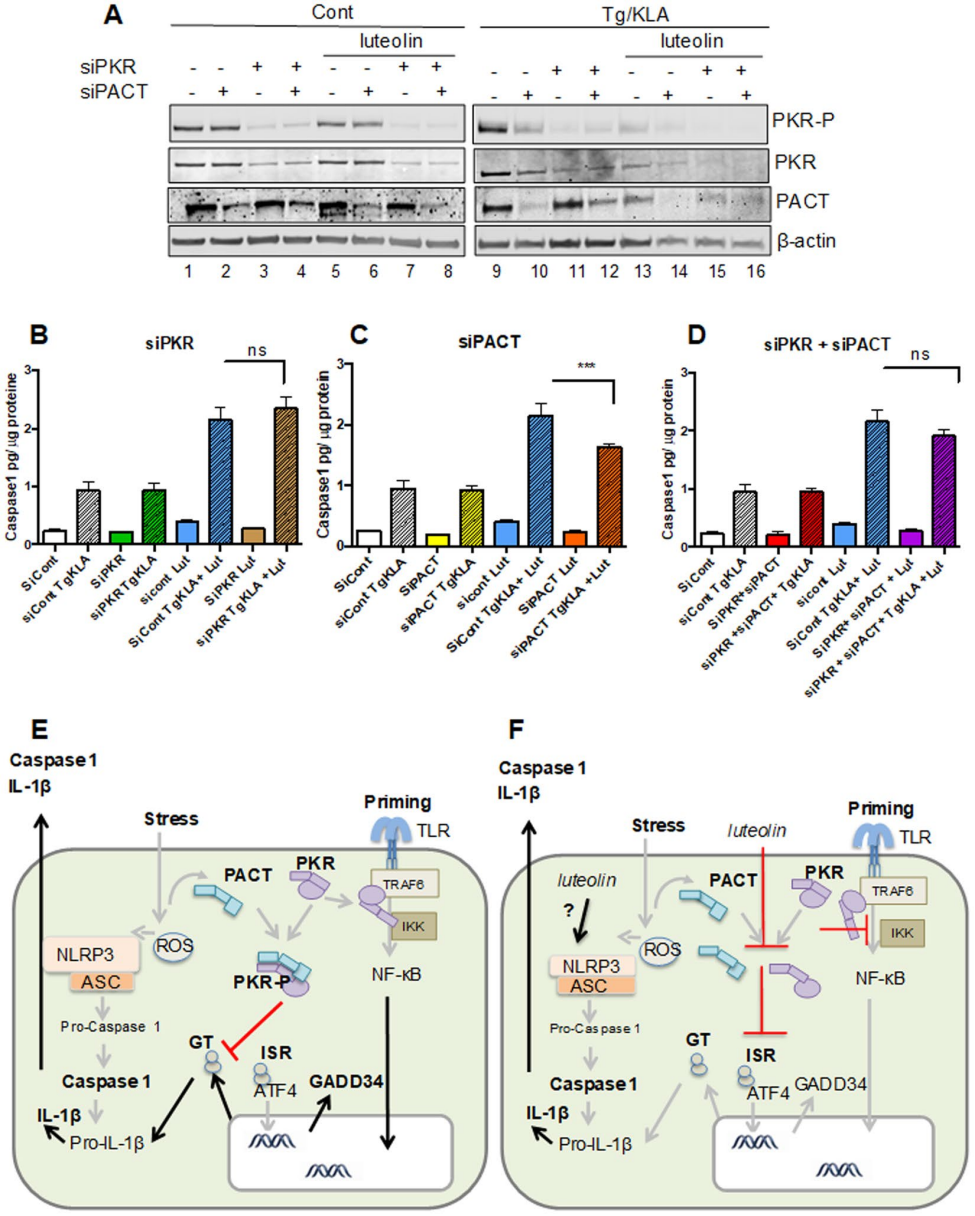
PKR or PACT are not involved in the activation of the inflammasome by luteolin. The THP1 cells were plated on wells containing 50 nM of siRNAcont, siRNA PKR, siRNA PACT, or both siRNA PKR and siRNA PACT, before addition of PMA. 24 hrs after, they were submitted to a second treatment with the siRNAs and either untreated or treated, after 72 hrs, with 50 μM of luteolin. After 30 min, incubation was continued as such or in the presence of 5 μM of thapsigargin and 100 ng/ml KLA for 8 hrs. (**A**) Lysates were prepared and proteins were separated by SDS-PAGE prior to fluorescent immunoblot analysis of PKR and PACT. Detection of β-actin served as control (**B**,**D**). Activation of Caspase1 in the supernatants was determined by ELISA and the values were normalised to the amount of total protein for each condition. Data represents means ± SD from three independent supernatants. (**E**,**F**) *Graphical model* Activation of ROS (Reactive Oxidative Stress) in response to a stress leads to dimerization of PACT, its association with PKR, dimerization and activation of PKR as a kinase (PACT and PKR are represented here only as monomers for clarity). PKR inhibits general protein translation (GT), while turning on an integrative stress response (ISR), in which the favoured translation of transcription factor ATF4, allows restoration of homeostasis, through induction of specific genes, such as GADD34. Activation of the NF-κB signalling pathway, in response to specific TLR agonists (Priming), triggers induction of pro-inflammatory cytokines such as IL-8 (not shown here) and pro-IL-1ß. PKR can participate in this NF-κB activation as a scaffold protein, through binding to TRAF6 via its C-terminus and binding to the IKK complex via its DRBDs, independently of its interaction with PACT. The conjugation of both priming and ROS-induced events trigger the inflammasome (complex NLRP3/ASC), leading to cleavage of pro- Caspase1 and pro-IL-1ß and their secretion (**E**). Luteolin dissociates the PKR/PACT interaction, inhibits PKR phosphorylation and the IRS (since ROS activates also the IRS through PERK, the latter inhibition is not complete). Luteolin also inhibits induction of pro-inflammatory cytokines, at least in part through its ability to interfere with the DRBDs of PKR. In contrast, luteolin activates the inflammasome (through a still unknown mechanism,) in a PKR and PACT-independent manner, which rules out a role for PKR in the control of the inflammasome (**F**).

We have shown that luteolin disrupts the PKR/PACT interaction and behaves as an inhibitor of integrative stress response. We have shown also that luteolin inhibits the induction of pro-inflammatory cytokines but that it could enhance activation of the inflammasome. We next checked whether PKR or PACT could be responsible for this effect, in a kinase-independent manner. For instance, their dissociation in the presence of luteolin might have favoured association of PKR or PACT as monomers with some components of the inflammasome. The THP1 cells were silenced for either PKR or PACT using siRNAs and submitted to thapsigargin/KLA treatment in the presence or not of luteolin. An immunoblot analysis first confirmed the ability of luteolin to inhibit PKR phosphorylation in response to thapsigargin/KLA (Fig. 8A; compare lane 13 to 9). In addition, these data revealed that silencing of PACT also inhibits activation of PKR by thapsigargin/KLA and that this effect is aggravated in the presence of luteolin (Fig. 8A; compare lane 10 to 9 and 14 to 13). This confirms the role of PACT and of its interaction with PKR to trigger PKR activation, in response to stress. Silencing of either PKR or PACT were indicated by significant inhibition of expression levels of these proteins (Supplemental Fig. 8A). In regards to the effect of the depletion of PKR or PACT on the activation of the inflammasome, we found that PKR or PACT, either silenced separately or together, had no influence on the activation of caspase 1 by the thapsigargin/KLA treatment, (Fig. 8B–D). Furthermore, the increase in the activation of caspase1 by luteolin was not affected by silencing PKR or PACT alone, or PKR and PACT together. Only a slight inhibition was observed by PACT silencing (Fig. 8C). Altogether, these data show that activation of inflammasome by luteolin may involve interaction with partners independent on its action on PKR and PACT and that PKR or PACT are not participating in activation of the inflammasome.

## Discussion

In this study, we performed a high-throughput screening to identify compounds that target the stress kinase PKR at the level of its activation by PACT, rather than at its catalytic domain. Our screening procedure was based on *in vitro* HTRF assay between tagged purified preparations of the DRBD-containing N-terminus PKR and full length PACT. The selected hits were further assayed for their ability to interfere with the interaction between PKR and PACT in a cellular context, using a split-Nanoluc protein complementation assay. This led to the identification of three members of the flavonoid family: myricetin, quercetin and luteolin. Using luteolin as a representative of this chemical series, we then demonstrated that inhibition occurs at the level of interaction between the DRBDs, whether in the homo-or hetero-dimerization configuration. In other words, those compounds are able to inhibit the PKR/PKR or the PACT/PACT interaction in addition to inhibit the PKR/PACT interaction. These data reveal for the first time that the PKR/PACT association can be specifically disrupted by members of the flavonoid family. Luteolin proved to be more efficient than the two other flavonoids in a subsequent PKR phosphorylation assay following an oxidative stress and its mechanism of action was further evaluated.

Luteolin belongs to the group of natural flavonoids, or polyphenols, that is abundant in vegetables, fruits and medicinal herbs. The plants rich in luteolin, such as Chrysanthemum flowers have been long known in Chinese traditional medecine for their anti-inflammatory effects and anti-cancer effects^27^. However, luteolin has pleotropic effects and its exact mechanism of action remains ill-defined. Luteolin is generally referred to as an anti-oxidant, or ROS scavenger, either through its own oxidation or through the inhibition of ROS-generating oxidases or by protecting endogenous anti-oxidant enzymes. In addition to this direct anti-oxidant properties, luteolin was also reported to inhibit LPS-induced activation of NF-κB and MAPK pathways, but its mode of action was not understood^27^. Luteolin is blood-brain barrier permeable and was reported to have a neuroprotective effect in drug-induced Alzheimer’s rat model^38^. These properties would be therefore in line with a role for PKR in impairment of cognition through inflammation. At present, there are only few clinical assays to assess the beneficial effects of members of the flavonoid family. A combination of luteolin and quercetin proved effective in reducing symptoms of autism spectrum disorders (ASD), which might be associated with inflammation in brain regions related to cognitive function^39^. Our study reveals that luteolin can inhibit two of the known functions of PKR: triggering the integrative stress response and its participation in the induction of NF-κB-dependent pro-inflammatory cytokines but, in contrast, was increasing the activity of the inflammasome, in a PKR (and PACT)-independent manner. A schematic representation of the inflammatory pathways at play in response to oxidative stress, and TLR activation is shown in Fig. 8E and the role of luteolin on these pathways is shown on Fig. 8F. The clear ability of luteolin to abrogate PKR/PACT association and PKR/PKR dimerization provided therefore a different approach to appreciate the role of PKR in mechanisms leading to inflammation, particularly in view of previous contradictory reports. For instance, it has been shown that murine macrophages either deficient for PKR due to a deletion in its catalytic domain^40^, or treated with 2-aminopurine (2AP) or the C16 compound presented inhibition of caspase 1 and production of IL-1β in response to DAMPs (Danger Associated Molecular Patterns)^4^. PKRwt, but not a catalytically inactive PKR point mutant was then shown to physically interact with NLRP3 which led to the conclusion that PKR interacts with NLRP3 and that its activity were required to activate the inflammasome^4^. In another study using macrophages from either the same PKR deficient mice or other PKR deficient mice through deletion of the first two exons of PKR^41^, no difference was observed in the activation of caspase 1 and the production of IL-1β after treatment of cells with LPS and DAMPs, leading to the opposite conclusion that PKR was not involved in the regulation of the inflammasome^8^. Compound 7DG isolated from a systematic search for inhibitors of the lethal anthrax toxin-activation of inflammasome by library screening, was reported to inhibit events leading to activation of caspase1 as well as interacting with the C-terminal half of PKR, without affecting its catalytic activity. Assembly of the inflammasome was inhibited in the presence of 7DG but not in the presence of 2AP or C16. The conclusion of the authors was that PKR was involved in inflammasome assembly but ruled out its role as a kinase in this process^6^. Recently, a novel model of PKR inactive mice has been generated in which PKR kinase activity was knocked-in by point mutation. The use of peritoneal macrophages either from these mice or from PKR deficient mice showed an increase in the formation of inflammasome (ASC nucleation), in the activity of caspase1 and secretion of IL-1β and IL-18 after cotreatment with LPS and DAMPs. The authors concluded that PKR exerts a negative control on the inflammasome at the level of protein translation, allowing to repress the induction of factors that are critical for the activity of the cryopyrin inflammasome^5^. NLRP3, as component of the inflammasome, is an important sensor of altered homeostasis and its deregulation is linked to a number of metabolic diseases. In particular, NLRP3 appears to be involved in obesity-induced inflammation and Type 2 diabetes. For instance, NLRP3-deficient mice are insulin hypersensitive when submitted to high fat diet (reviewed in^42^). Some studies have indicated a role for PKR in obesity-induced inflammation and Type 2 diabetes^43^ but again this has been questionned^44^.

Our results show that activation of the inflammasome is increased in the presence of luteolin that affects the homo- or heterodimerization of PKR and PACT but that this effect can not be abrogated when the expression of PKR or PACT is inhibited through silencing. Therefore, our data support the notion that PKR would not play an essential role in the control of the inflammasome. The use of luteolin, following its identification as disruptor of the PKR/PACT interaction and as a novel inhibitor of PKR allowed us also to show its ability to inhibit the induction of pro-inflammatory cytokines and we suggest that this might be, at least in part, to its ability to bind the DRBDs of PKR and interfere with the interaction of PKR with the NF-κB pathway. Our data support the notion that it could be beneficial for the organism to inhibit activation of PKR as a kinase in response to stress. Indeed, this would attenuate unwanted translations of mRNAs, as part of the integrative stress response, such as translation of BACE1 with subsequent secretion of amyloid-beta. PKR is also known to trigger activation of c-Jun N-terminal kinase (JNK) and both kinases are activated in the brains in the case of Alzheimer disease and are involved in Aβ production, neuroinflammation, and neuronal death. Recently, we have shown that a dual inhibition of PKR and JNK could nearly abolish Aβ toxicity in primary cultured neurons and would therefore mediate neuroprotection^45^. Similarly, thiamine deficiency, which leads to neuronal death, activates the PKR-eIF2α pathway and increases the BACE1 expression levels of Aβ in specific thalamus nuclei. This effect could be reversed by PKR downregulation^46^.

In conclusion, the anti-oxidant luteolin has been identified here as a novel inhibitor of PKR, by preventing PKR activation through its association with PACT and preventing also homodimerization of PKR. Luteolin can inhibit both the integrative stress response and induction of pro-inflammatory cytokines, two effects related to kinase and non-kinase functions of PKR, but can also increase activation of the inflammasome in a PKR-independent manner. This indicates that the use of compounds like luteolin in inflammatory-related diseases might be taken with caution and should be associated with direct anti-NLRP3 agents, such as the recently described MCC950^47^, to have the safest and more efficient outcome in patients.

## Materials and Methods

### Reagents

Thapsigargin was obtained from Enzo Life Sciences. The TLR4 agonist KLA (Kdo2-Lipid A) was from Santa Cruz Biotechnology. Sodium arsenite, the oxindole-imidazole-C16, salubrinal, the three flavonoids luteolin, myricetin and quercetin were from Sigma. The PERK inhibitor GSK2606414 (Perki) was from Calbiochem. The PKR and PERK inhibitors, the flavonoids and salubrinal were all kept in DMSO as stock solution then diluted in cell culture medium immediately before use. The CHAPS detergent (3-((3-cholamidopropyl) dimethylammonio)-1-propanesulfonate was from Sigma. Control siRNA (ON-TARGETplus Non-targeting Pool #D-001810-10-0X) and siRNA against PKR (ON-TARGETplus EIF2AK2 siRNA LQ-003527-00-0002) were from Dharmacon Research, Inc. (Lafayette, CO). siRNA against PACT (PRKRA, ID:s16335, Ambion; Life Technologies) was a kind gift of A. Komarova (Institut Pasteur).

### Cell culture

HEK-293T cells and Huh7.25/CD81 cells^48^ were cultured in Dulbecco’s modified Eagle’s Medium (DMEM + GlutaMAX; Gibco laboratories; Grand Island, NY, USA) supplemented with 10% heat-inactivated fetal bovine serum (FBS) (Hyclone; GE Healthcare Life Sciences), 1% nonessential amino acids (Gibco),1000 U/ml penicillin and 0.1 mg/ml streptomycin (Invitrogen; USA). The human monocytic leukaemia cell line THP1 was cultured in RPMI + Glutamax (Gibco) supplemented with 10% FBS and penicillin/streptomycin as above. The THP1 cells were differentiated in macrophages by treatment with 100 ng/ml PMA (phorbol 12-myristate 13-acetate; Sigma) for 72 hrs, after being plated at 2 × 10^6^ cells/well in 12-well plates.

### Expression vectors and antibodies

The pGEX-4T vector expressing the PKR 1–265 fragment in fusion with GST at its N-terminus (GST-PKR Nter) and the pET15b vector expressing the PACT protein have been previously described^2,15^. The pENTR™-SD⁄D-TOPO® vector and the pDEST24 were purchased from Invitrogen. Normal mouse or rabbit IgGs were from Santa Cruz Biotechnology. Mouse monoclonal anti-PKR 71/10 antibody was produced by Agrobio Laboratories (FR) as reported^49^. Mouse monoclonal Anti-ß-actin antibody was from Sigma (A1978). Rabbit polyclonal antibodies were used to detect human PKR phosphorylated at T446 (ab32036; Abcam). Anti-PACT (sc-377103) and anti-NFκB p65 (sc-109) polyclonal antibodies were purchased from Santa Cruz Biotechnology. Goat anti-Rabbit IgG (H&L) Secondary Antibody, DyLight 800 4X PEG (#SA5-35571), Goat anti-Mouse IgG (H + L) Secondary Antibody, DyLight 680 (#35518) and Alexa Fluor 488 goat anti-rabbit IgG secondary antibody (A11034) were from Invitrogen.

### Immunofluorescence

Cells were fixed in PBS containing 4% paraformaldehyde (PFA) for 20 min in room temperature and permeabilized with PBS containing 10% FBS and 0.3% Triton X-100. Non-specific antibody sites were blocked with PBS containing 2.5% BSA, 10% FBS for 30 min at room temperature. For staining, cells were incubated with antibodies diluted in this blocking solution. Primary antibodies were added for 1 hr and secondary antibodies were added for 30 min in room temperature. Nuclei were visualized by incubation with DAPI for 5 min at room temperature.

### Confocal imaging

Confocal fluorescence microscopy studies were performed with a Zeiss Axiovert 100-M inverted microscope equipped with a LSM 700 laser scanning unit and a 1.4 NA × 63 Plan-Apochromat oil-immersion objective. Cells were seeded in μ-Slide 8 well, ibiTreat, tissue culture treated, (Inter Instrument AS). To minimize photobleaching, laser power was typically 20% under maximum and the pinhole was set to 0.8–1.5. Multi-tracking was used for dual color imaging.

### Large-scale production and purification of GST-PKR and His-PACT

*Escherichia coli* BL21-CodonPlus(DE3), transformed with pGEX-4T (GST-PKR Nter) or pET15b (His-PACT) were grown in high density medium in micro-fermentors at 30 °C. Induction of GST-PKR Nter or His-PACT expression was performed by adding 1 mM isopropyl-1-thio-β-d-galactopyranoside (IPTG; Sigma) for an additional 2 hrs of incubation at 30 °C (PKR) or 0.01 mM IPTG for 24 hrs at 30 °C (PACT). The bacteria pellets were stored at −80 °C. Bacteria pellets were resuspended in Phosphate-Buffered Saline, pH7.5 (PBS) containing 1% Triton X-100, 1 mM DTT, anti-proteases (Roche) and benzonase (Sigma) for PKR or 20 mM Tris-HCl pH 7.9, 200 mM NaCl, 5 mM imidazole, containing anti-protease and benzonase for PACT. The pellets were homogenized in a blender (Waring Commercial), submitted to cell desintegrator at 2.7 KBar (Constant System TS), adjusted to 2 mM PMSF and centrifuged at 43,000 g for 1 hr at 4 °C in a SS34 rotor of a Sorvall centrifuge. For purification of GST-PKR Nter, 60 ml of the soluble fraction were loaded on 5 ml of Protino® Glutathione Agarose 4B (Macherey-Nagel) using ÄKTA pure chromatography system at 4 °C. GST-PKR was eluted in Tris-HCl pH 8.5, 1 mM DTT, 20 mM Glutathione. After profile analysis by SDS-PAGE, the selected fractions were pooled, concentrated on Vivaspin 20, dialysed against Tris-HCl, pH8.5, 150 mM NaCl and the purified protein was stored at 1 mg/mL at 4 °C. For purification of His-PACT, the insoluble fraction was washed twice with 20 mM Tris-HCl pH 7.9, 200 mM NaCl, 5 mM imidazole followed by centrifugation at 43,000 g for 30 min at 4 °C. The pellet was then resuspended in denaturation buffer (20 mM Tris-HCl pH 7.9, 200 mM NaCl, 6 M urea), rotated overnight at 4 °C and centrifuged at 43140 g for 60 min at 20 °C. The supernatant was loaded on a 1 ml Protino Ni-NTA (Macherey-Nagel) column using ÄKTA Express chromatography system at room temperature. After washes, first in equilibration buffer and then in equilibration buffer containing 60 mM imidazole, the protein was eluted with 20 mM Tris-HCl pH 7.5, 200 mM NaCl, 6 M urea, 100 mM EDTA. After profile analysis by SDS-PAGE, the selected fractions were pooled and dialyzed against 20 mM Tris-HCl, pH 7.5, NaCl 200 mM at 4 °C in four steps of decreasing urea concentrations (4, 2, 1 and 0.5 M, and finally no urea) using dialysis cassettes (Thermo Slide-A-Lyser G2 3500 MWCO; Thermo Fisher Scientific). The purified renatured protein was stored at 4 °C. For PKR or PACT, the possible presence of protein aggregates was measured by Dynamic Light Scattering (DLS).

### Screening of compound library by HTRF assay

A chemical collection composed of 39,373 compounds from commercial libraries acquired from Prestwick Chemical (1,200 compounds; www.prestwickchemical.com) and CHEM-X-INFINITY (10,000 compounds; www.chem-x-infinity.com) and from the French academic chemical library called “Chimiothèque Nationale”^50^ (28,173 compounds obtained from Institut de Chimie et Biochimie Moléculaires et Supramoléculaires (ICBMS) de Lyon, Faculté de Pharmacie de Strasbourg, Centre d’Etude et de Recherche sur le Médicament de Normandie (CERMN), Institut Curie and Institut Pasteur) was screened on a Freedom EVO^®^ platform (Tecan). Compounds from Prestwick Chemical and CHEM-X-INFINITY were at 2 mg/ml (average concentration of 6.32 ± 2.8 mM), and 10 mM respectively, and those from the “Chimiothèque Nationale” were at the following concentrations for the different subsets: ICBMS at 10 mM, Faculté de Pharmacie de Strasbourg at 2 mg/ml (7.43 ± 2.48 mM), CERMN at 3.3 mg/ml (10.5 ± 3.35 mM), Institut Curie at 2 mg/ml (7.34 ± 2.76 mM), Institut Pasteur either at 3.3 mg/ml (12.8 ± 3.71 mM) or 2 mg/ml (6.81 ± 2.13 mM). All compounds were screened at a 1:80 dilution and a final DMSO concentration of 1.25% (v/v). The HTRF assay was performed into white, small volume, bar-coded tissue culture 384-wells plates (Greiner Bio-One). For each plate, columns 1, 2, 23 and 24 were dedicated to negative (8 wells with GST-PKR-Nter alone and 8 wells with His-PACT alone) and positive (16 wells) controls, where the same amount of DMSO was applied. 1 µL of compound in DMSO solution was loaded into dry wells from columns 3 to 22. Then, 5 µl of GST-PKR Nter (200 nM in Tris-HCl 20 mM pH7.5, NaCl 200 mM) and 5 µL of His-PACT (266 nM in Tris-HCl 20 mM pH7.5, NaCl 150 mM) were added sequentially to all wells, except for the negative controls where either GST-PKR-Nter or His-PACT alone (8 wells each) was added. After a 1 hr incubation at 30 °C, 9 µL of a mix of Anti-GST-XL665 (33.3 nM) and Anti-6HIS-Tb (3.11 nM) conjugates (Cisbio) were added. After 24 hrs of incubation at RT, the fluorescence emission was measured at 620 nm and 665 nm using an excitation wavelength at 340 nm on microplate reader (Infinite M1000 Pro (Tecan)). The HTRF ratio was defined as following: Ratio = (Fluorescence intensity at 665 nm/Fluorescence intensity at 620 nm) × 10^4^. Data were converted from HTRF ratio to ΔF % via the following equation: ΔF % = [(Sample HTRF ratio of the compound − Ratio_neg_)/Ratio_neg_] × 100, where Ratio_neg_ is the HTRF ratio value of the negative controls with only GST-PKR-Nter. For each plate, the Z’-factor^51^ was calculated as following 1–3 × (standard deviation of the positive controls + standard deviation of the negative controls)/(mean of the positive controls – mean of the negative controls). Average Z′-factor was determined to be 0.569 ± 0.08 and the average fluorescence ratio between the positive and negative controls was 6.5 ± 1.2. The IC_50_ was calculated using PRISM version 5.0 software (GraphPad Software Inc, CA, USA) from the non-linear curve fitting of the ΔF % value versus the inhibitor concentration, using the same HTRF assay (measurement on an EnVision (Perkin Elmer)) set up for the screening campaign.

### Measurement of cytotoxicity

Huh7.25-CD81 were seeded in 96-well culture plates at a density of 5 × 10^4^ cells and incubated for 5 h to allow cell attachment to the plates before the addition of the chemical coumponds in the concentration range of 400–0.8 µg/ml. After 18 h, cytotoxicity was determined by the “methyl-thiazol-tetrazolium assay” (MTT) according to the manufacturer’s instructions (Sigma). Cell viability was obtained as percentage of the negative control group, which represented 100% cell viability.

### ELISA

Human Caspase-1 and IL-1β secreted by THP1-derived macrophages were quantified by ELISA using Quantikine ELISA to hu-caspase-1 kit (R&D Systems, ref. DCA100) and hu-IL1β/IL1-F2 kit (R&D System, ref.DY201), respectively. Both kits were used as recommended by the manufacturer.

### Immunoblot

Cells were washed once with PBS and scraped into CHAPS buffer (50 mM Tris-HCl pH 7.5, 140 mM NaCl, 5 mM EDTA, 5% glycerol, 1%CHAPS) that contained phosphatase and protease inhibitors (Complete, Roche Applied Science). The protein concentration was determined according to Bradford. Protein electrophoresis was performed on NuPAGE 4–12% Bis TRIS gels (Invitrogen). Proteins were transferred onto nitrocellulose membranes (Fisher Bioblock Scientific), and probed with specific antibodies as described in Materials and methods and in the figure legends. Fluorescent immunoblot images were acquired and quantified by using an Odyssey scanner and the Odyssey 3.1 software (Li-Cor Biosciences).

### Quantative real-time PCR analysis

Total cellular RNA was extracted using TRI-Reagent (Sigma), according to the manufacturer’s instruction (Sigma). Host cell mRNAs expression was quantified by a two-step qRT-PCR assay. A list of the different primers used is provided in Supplementary Table 1. The reverse transcription step was performed on 1 µg of total RNA. Quantitative real time PCR was performed using an AbiPrism 7900HT machine, with a FastStart Universal SYBR Green Master (Roche). The results were normalized to the amount of GAPDH cDNA.

### Sensing protein-protein interactions by protein complementation assay

The ORF sequences corresponding to PKR-Nter 1–265 and full length PACT were cloned by *in vitro* recombination into pDONR207 (BP cloning reaction; Invitrogen). Then, the PKR-Nter and PACT from the pDONR207 were transferred by recombination into vectors expressing complementary fragments of Nanoluc luciferase, to be in fusion with these luciferase domains, either at their N- (Luciferase N1 or N2) or C- (Luciferase C1 or C2) terminus^29^. The protein complementation assay was performed in 96-well plates using HEK-293T cells (32,000 cells/well) or Huh7.25/CD81 cells (30,000 cells/well). After 24 hrs, cells were transfected with 100 ng of PKR-Nter or PACT plasmids bearing N1, N2, C1 or C2. Cells were treated with different concentrations of the chemical products. Treatment was done either at the time of transfection up to 6 hrs post transfection with identical effect on the luciferase activity. At 24 hrs post-transfection, the medium of the cells was harvested and the cells incubated in the presence of 50 μl of Nano-Glo luciferase reagent (Promega #N1150). Luciferase enzymatic activity was measured using a Centro XS LB960 luminometer (Berthold) using a 5 s integration time. For each interaction, values were calculated from the mean of triplicate experiments.

### Preparation of microglia

Animal experimental procedures were performed according to the french regulations. The project was approved by the Animal Ethics Committe of the University of Paris-Diderot, Paris, France (Lariboisiere/Villemin: 89932016062110581948 V3). Two pregnant C56BL6 females at 15 days of embryonic age were used as donors of the embryos. Mice were anaesthetized and cervical dislocation was performed. The embryos (total number of 14) were removed and placed in a saline solution (PBS Ca^2+^ free, Mg^2+^ free) containing 0.6% glucose. Hippocampal tissues were taken in sterile conditions under a microscope and placed in the same buffer and dissociated by trypsin (2.5 mg/mL trypsin in PBS) for 20 min at 37 °C. Reaction was stopped using cotreatment with inhibitors of DNAse (DN25, 0.25 mg/mL; Sigma) and trypsin (T9128, 1.6 mg/mL; Sigma) in DMEM (#31885; Gibco). The cells (astrocytes and microglia) were transferred at 3.5 × 10^6^ cells/ dish in DMEM FCS 10% culture media in 35 mm petri dishes coated with a solution of a 1/10 dilution of Poly L-ornithine (P4957; Sigma) in RNAse free water. They were incubated for 15 days with a change of medium at day 7. At day 15, microglia was separated by tapping from the astrocytes, which remained attached to the dishes and transferred to novel Poly L-ornithine-coated dishes.

## Electronic supplementary material

Supplementary Information
