# Supplementary material for: Inhibition of the inflammatory response to stress by targeting interaction between PKR and its cellular activator PACT
Source: Sci Rep. 2017 Nov 23;7:16129. doi: 10.1038/s41598-017-16089-8 (PMC5701060; doi:10.1038/s41598-017-16089-8)
Supplement: Supplementary file 1 — Supplementary Information [file 41598_2017_16089_MOESM1_ESM.pdf]

Inhibition of the inflammatory response to stress by targeting interaction between PKR and its cellular activator PACT

Stephanie Dabo[1,2], Patrick Maillard[1,2], Milagros Collados Rodriguez[1,2], Marianne Doré Hansen[1,2,3], Sabrina Mazouz[1,2], Donna-Joe Bigot[1,2], Marion Tible[4,5], Geneviève Janvier[1,2], Olivier Helynck[6,7], Patricia Cassonnet,[2,8], Yves Jacob[2,8], Jacques Bellalou[9], Anne Gatignol[10], Rekha C.Patel[11], Jacques Hugon[4,5], Hélène Munier-Lehmann[6,7] and Eliane F. Meurs[1,2,*]

[1]*Unité Hepacivirus and Innate Immunity, Institut Pasteur, 75015 Paris, France*

[2]*CNRS, UMR 3569,* Paris, France

[3] *Department of Clinical and Molecular Medicine, Faculty of Medicine and Health Sciences,Norwegian University of Science and Technology, 7006 Trondheim, Norway*

[4] *Center of Cognitive Neurology, Lariboisière Hospital AP-HP University Paris Diderot, 75010, Paris, France*

[5] *Inserm U942,Paris, France*

[6] *Unité de Chimie et Biocatalyse, Institut Pasteur, 75015 Paris, France*

[7] *CNRS, UMR3523, Paris, France*

[8] *Unité de Génétique Moléculaire des Virus à ARN, Institut Pasteur, Université Paris Diderot, Paris, France*

[9]*Plate-forme des protéines recombinantes, Institut Pasteur, 75015 Paris, France, CNRS UMR 3528*

[10]*Virus-Cell Interactions Laboratory, Lady Davis Institute for Medical Research, Department of Medicine, department of Microbiology and Immunology,McGill University,Montreal, Quebec*

[11]*University of South Carolina, Department of Biological Sciences, Columbia, South Carolina 29208, USA*

Correspondence to : eliane.meurs@pasteur.fr

Supplementary Table 1 : Primers used for RTqPCR analysis

Supplementary Table 2 : Data for the hits selected through the screening procedure.

From the 37 compounds of the Prestwick Chemical Library® that inhibited the PKR/PACT interaction by more than 60% (<40% binding) during the screening procedure, we discarded those which were toxic, could not pass the blood-brain barrier, used only topically or irrelevant in regards to a role for PKR in stress, according to published information. In the remaining list, we have chosen 5 compounds from highest to lowest efficiency on PKR/PACT binding, within the 0-40% range. Those were myricetin, gossypol, ergocalciferol, quercitin and moroxydine (2.1%, 7.7%, 16.3%, 22.6%, 38.1% PKR/PACT binding remaining after incubation with compounds, respectively). For the other libraries, we have selected within the same range (0-40 %) 46 compounds among the 1,263 hits from the "Chimiothèque

Nationale" and 30 compounds among the 177 hits from the CHEM-X-INFINITY library. Those were chosen among the hits giving the highest inhibitory potency of PKR/PACT interaction and according to their availability. Three other rounds of HTRF assays were then performed. In the first one, serial dilutions of the compounds were incubated with GST-PKR (100 nM), His-PACT (100 nM) and the anti-6His Lumi4Tb (4nM) and anti-GST XL665 (15 nM) antibodies. This assay was used to determine the IC_{50} (inhibition PKR/PACT binding), calculated as described in Materials and Methods. In parallel, their cytotoxicity have been also assessed by the MTT assay. In the two other assays, HTRF assays were performed to discard compounds with non specific inhibition effect, i.e, those which inhibit the fluorescence from the pairs GST-XL665 conjugate/anti-GST-Cryptate or 6 His- XL665 conjugate/anti-6His Cryptate (GST check kit and 6His Check kit Gold ; Cisbio) (no target PKR or PACT). After these rounds of selection, 2 compounds were kept from the Prestwick Chemical Library®, 14 from the French Academic chemical library and 1 from the Chem-X-Infinity library. A split luc assay in HEK 293T cells was then performed to evaluate the potency of the compounds to dissociate the PKR/PACT complex in a cellular context. In parallel to this, the compounds were assayed for their ability to inhibit PKR phosphorylation in THP1 macrophages submitted to sodium arsenite treatment, as described in figure 3. Luteolin was selected as the compound, which fulfilled all criteria.

Suppl Figure 1 B : **Full length gels showing purified preparations of PKR and PACT.**
Analysis of the GST-PKR-Nter and His-PACT proteins by SDS-PAGE after purification on glutathione-sepharose and on Ni-charged His-bind resin, respectively. Migration was performed on 12% gel Criterion (BioRad) in MES SDS running buffer (Life Technologies). Coomassie staining of gels showing the profile of GST-PKR-Nter and His-PACT after purification. For GST-PKR-Nter, lanes 1-3 represent total proteins before loading on glutathione-sepharose, the pass-through and wash. For His-PACT, lanes 1-2 represent total proteins before loading on Ni-charged His-bind resin and the pass-through. 3 represents two wash fractions and the first fraction of the elution step. The purified fractions were pooled and processed for resuspension as soluble proteins as described in Materials and Methods.

Suppl Figure 3. Full length gels showing PKR phosphorylation in THPI macrophages submitted to stress

A : The THP1 cells were incubated for the indicated times in the absence or presence of 50 µM sodium arsenite (NaArs). Lysates were prepared and proteins were separated by SDS-PAGE prior to incubation first with rabbit polyclonal anti- PKR-T446 antibodies and Goat anti-Rabbit IgG (H&L) Secondary Antibody, DyLight 800 4X PEG. Acquisition of the image was performed with Odyssey Imaging system (Li-Cor). The gel was then cut around 55 kda ; the upper part was incubated with mouse monoclonal anti-PKR 71/10 antibody, the lower part with mouse monoclonal Anti-ß-actin antibody and the two membranes were then incubated with Goat anti-Mouse IgG (H+L) Secondary Antibody, DyLight 680. The image was acquired as above. Conversion of the bands detected at 800 or 680 was converted to black and white with Photoshop.

C: The THP1 cells were either untreated or incubated for 30 min with C16 (400 nM) or different concentrations of myricetin, quercetin or luteolin, after which they were incubated in the presence of 25 µM NaArs. Lysates were prepared and proteins were separated by SDS-PAGE prior to incubation first with rabbit polyclonal anti-PKR-T446 antibodies and Goat anti-Rabbit IgG (H&L) Secondary Antibody, DyLight 800 4X PEG. Acquisition of the image was performed with Odyssey Imaging system (Li-Cor). The gel was then cut around 55 kda ; the upper part was incubated with mouse monoclonal anti-PKR 71/10 antibody and incubated with Goat anti-Mouse IgG (H+L) Secondary Antibody, DyLight 680, the lower part was incubated with anti-PACT rabbit polyclonal antibodies and mouse monoclonal Anti-ß-actin antibody and then incubated with Goat anti-Mouse IgG (H+L) Secondary Antibody, DyLight 680 and Goat anti-Mouse IgG (H+L) Secondary Antibody, DyLight 680. The image was acquired by Odyssee as above. Conversion of the bands detected at 800 or 680 was converted to black and white with Photoshop.

Suppl Figure 4A. Full length gels showing effect of Thapsigargin and KLA on PKR phosphorylation in THP1

The THP1 cells were either untreated or incubated for 8 hrs in the presence of 2 or 5 µM of thapsigargin (Tg), KLA (100 ng/ml) or both drugs (left), or in the presence of 25 µM sodium arsenate (NaArs), KLA (100 ng/ml) or both drugs (right). Lysates were prepared and proteins were separated by SDS-PAGE prior to fluorescent immunoblot analysis of PKR, phosphorylated PKR and β-actin with rabbit polyclonal anti-PKR-T446 antibodies, monoclonal anti-PKR 71/10 antibody and mouse monoclonal Anti-ß-actin antibody followed by incubation with Goat anti-Rabbit IgG (H&L) Secondary Antibody, DyLight 800 4X PEG or Goat anti-Mouse IgG (H+L) Secondary Antibody, DyLight 680 as described in Suppl Figure 3. Acquisition of the image was performed with Odyssey Imaging system (Li-Cor). Note that the lower part of the immunoblot on the left corresponding to the Tg/KLA treatment was also used to reveal the presence of PACT with anti-PACT rabbit polyclonal antibodies. This part of the blot is not shown in the manuscript.

Suppl Figure 8A. 1) Full length gels showing expression levels of PKR or PACT after silencing

The THP1 cells were plated on wells containing 50 nM of siRNAcont, siRNA PKR, si RNA PACT, or both siRNA PKR and siRNA PACT, before addition of PMA. 24 hrs after, they were submitted to a second treatment with the siRNAs and either untreated or treated, after 72 hrs, with 50 µM of luteolin. After 30 min, incubation was continued as such or in the presence of 5 µM of thapsigargin and 100 ng/ml KLA. After 8 hrs, lysates were prepared and proteins were separated by SDS-PAGE prior to fluorescent immunoblot analysis of PKR ,PACT and β-actin as described in Suppl Figure3C or 4A. Note that, because of the presence of a large spot on the membrane used to detect PACT, an other immunoblot was prepared with the same extracts.

2) Quantification of the efficiency of PKR and PACT silencing in the expt shown in Figure 8A.

Lysates from the experiment described in figure 8A were separated by SDS-PAGE prior to fluorescent immunoblot analysis of PKR ,PACT and β-actin as described in figure 8A to perform quantification of PKR and PACT after acquisition of the image by Odyssee. For each gel, the intensity of fluorescence of PKR and PACT in each band (delimited by blue squares) was first

normalized to the content of actin in each sample. The levels of the normalized PKR and PACT in lanes 2-8 are expressed after division by their level in lane 1 (right: no treatment) and those in lanes 10-16 after division by their level in lane 9 (left : treatment with Tg/KLA)

Name	Primer	Sequence
Human GAPDH	Forward	5’-ggtcggagtcaacggatttg-3’
	Reverse	5’-actccacgacgtactcagcg-3’
Human IL8	Forward	5’-aagggccaagagaatatccgaa-3’
	Reverse	5’-actagggttgccagatttaaca-3’
Human GADD34	Forward	5’-ggtcctgggagtatcgttca-3’
	Reverse	5’-cagggaggacactcagcttc-3’
Human IL1 ß	Forward	5'-acagatgaagtgctccttcca-3'
	Reverse	5'-grcggagattcgtagctggat-3'
Murine HPRT	Forward	5'-cgtcgtgattagcgatgatg-3'
	Reverse	5'-acagagggccacaatgtgat-3'
Murine IL6	Forward	5'-agttgccttcttgggactga-3'
	Reverse	5'-tccacgatttcccagagaac-3'
Murine IL1 ß	Forward	5'-gaccttccaggatgaggaca-3'
	Reverse	5'-agctcatatgggtccgacag-3'
Murine GADD34	Forward	5'-ctgcaaggggctgataagag-3'
	Reverse	5'-gctatggaagcagcagaagc-3'

Name of library	number of hits analysed	Number of specific hits	Name or Nr of compound	inhibition PKR/PACT by split luc assay	IC50 (µM) eff	IC50 (µM) cytotox	Inhibition PKR phosphorylation	other effects
Prestwick	5	2	Myricetin	YES	0,6	486	NO	
			Quercitin	YES	2	1083	*	
"Chimiothèque Nationale"	46	14	#16	NO	29	110	**	
			#17	nd	49	165	NO	
			#18	nd	58	37	*	
			#19	nd	25	201	NO	
			#24	nd	3	106	**	prot degrad
			#31	nd	25	48	*	
			#32	nd	4	150	*	
			#34	nd	3	74	**	
			#36	YES	40	224	**	
			#39	YES	26	80	*	
			#40	YES	16	79	*	
			#41	nd	0,2	184	*	
			#44/luteolin	YES	50	1397	****	
			#45	nd	30	1323	*	
Chem-X-Infinity	30	1	#61	NO	45	734	NO	Increase PKR-P

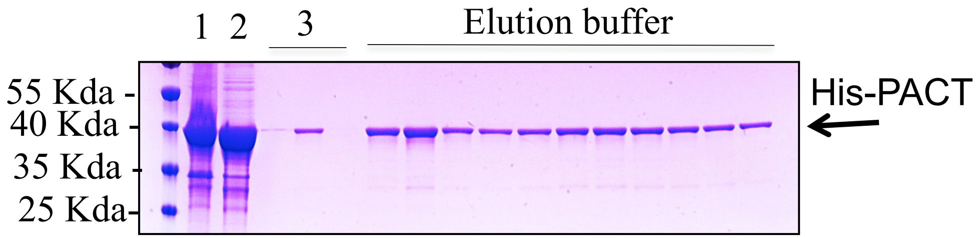
1
2
3
Elution buffer
55 Kda -
40 Kda -
35 Kda -
25 Kda-
His-PACT

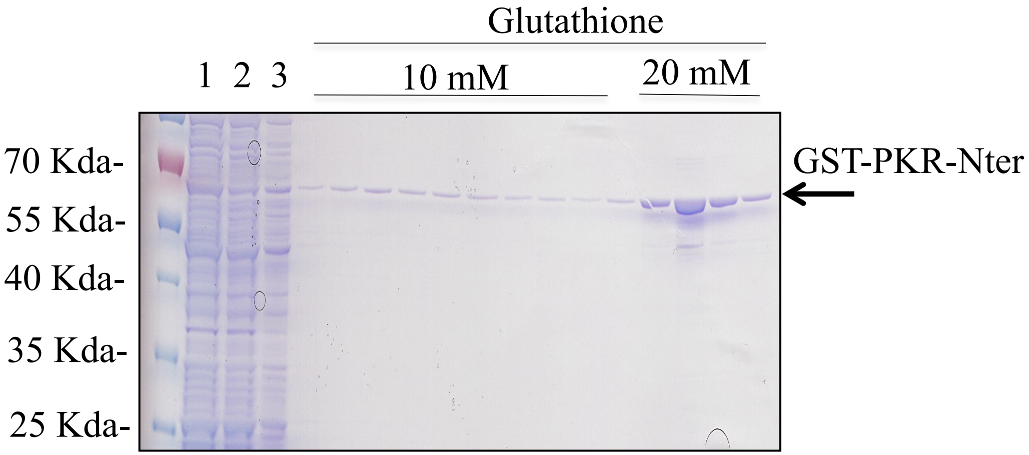
Glutathione
1 2 3
10 mM
20 mM
70 Kda-
55 Kda-
40 Kda-
35 Kda-
25 Kda-
GST-PKR-Nter

Figure 1B

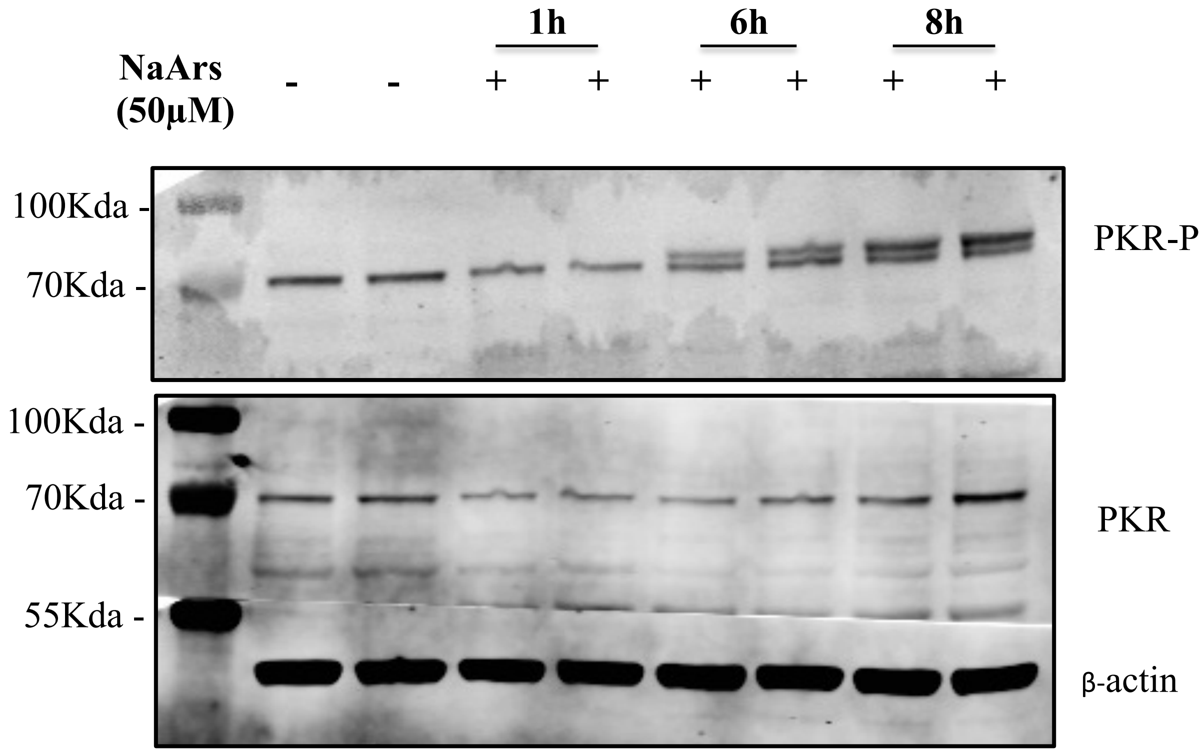
1h
6h
8h
NaArs (50µM)
- - + + + + + +
100Kda -
70Kda -
PKR-P
100Kda -
70Kda -
PKR
55Kda -
β-actin

Figure 3A

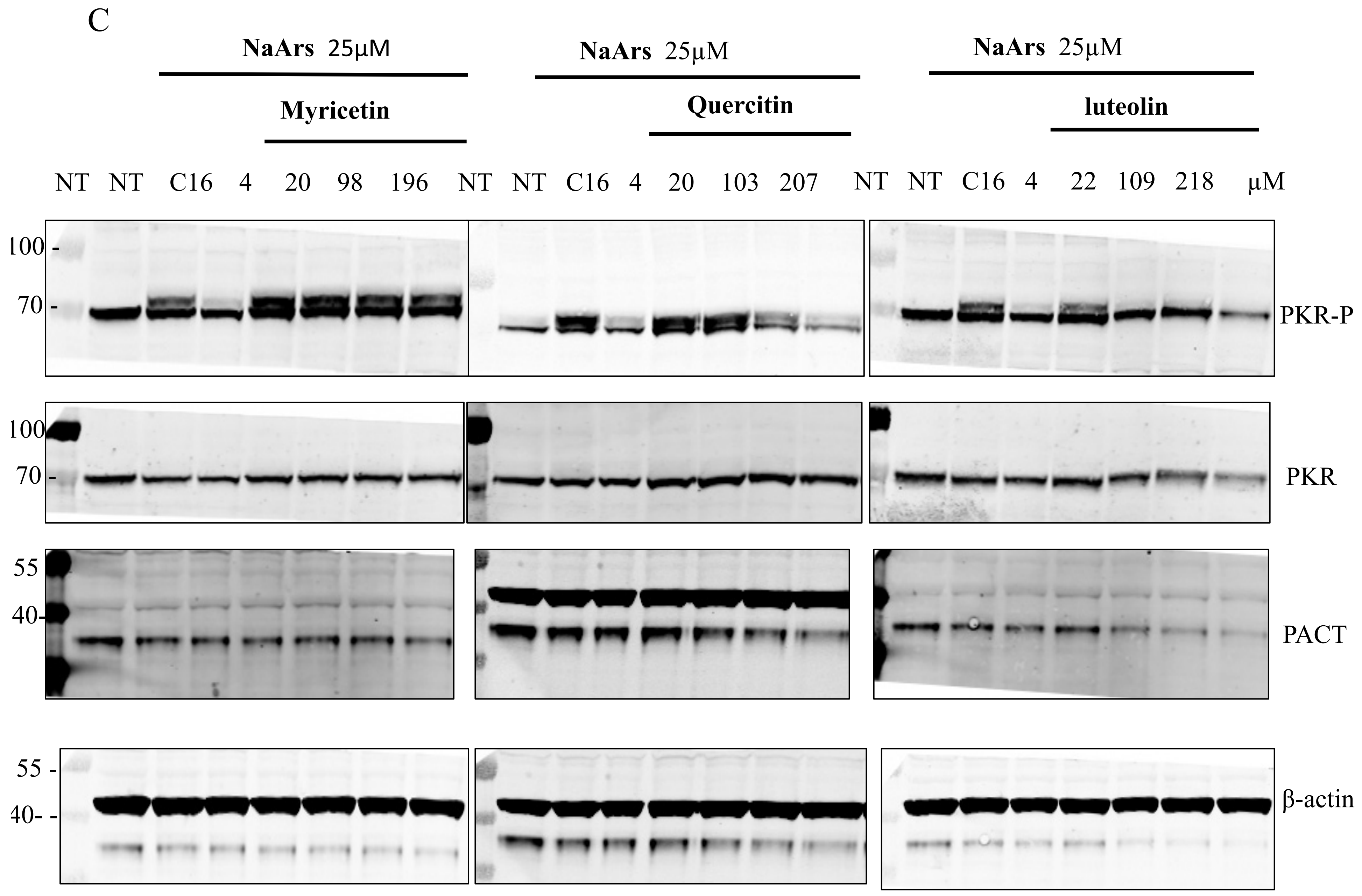
C
NaArs 25μM
Myricetin
NaArs 25μM
Quercitin
NaArs 25μM
luteolin
NT NT C16 4 20 98 196 NT NT C16 4 20 103 207 NT NT C16 4 22 109 218 μM
100 -
70 -
PKR-P
100
70 -
PKR
55
40-
PACT
55 -
40- -
β-actin

Figure 3C

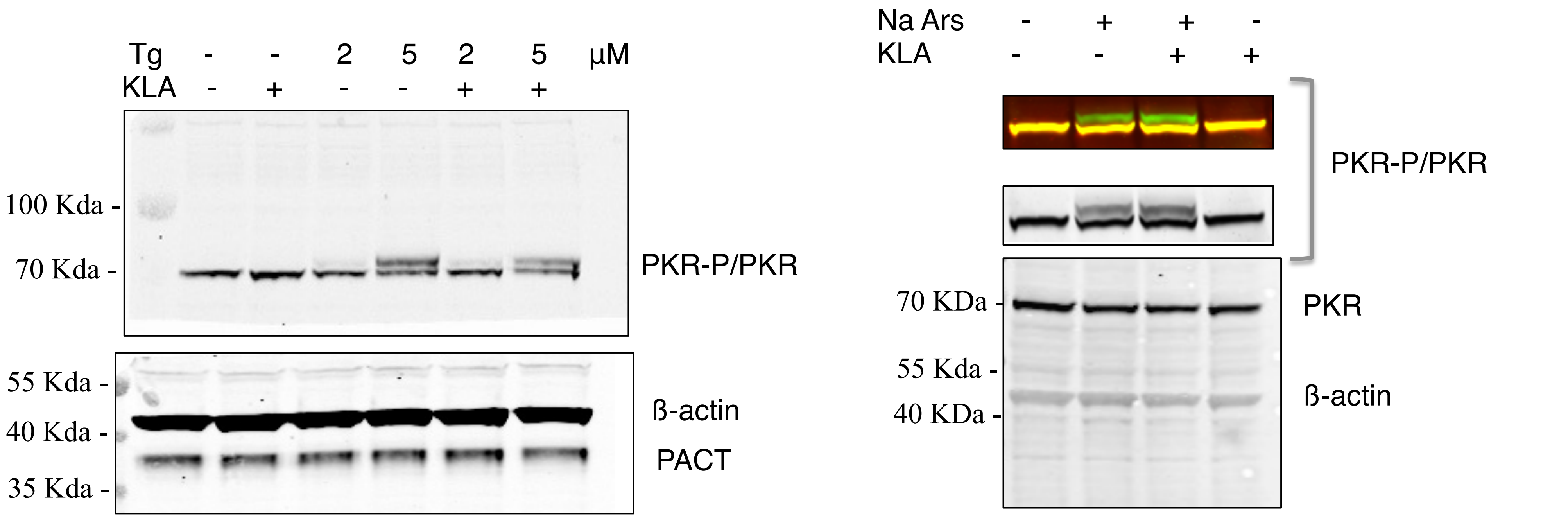
Tg - - 2 5 2 5 µM
KLA - + - - + +
100 Kda -
70 Kda -
PKR-P/PKR
55 Kda -
40 Kda -
35 Kda -
ß-actin
PACT
Na Ars - + + -
KLA - - + +
PKR-P/PKR
70 KDa -
PKR
55 Kda -
40 KDa -
ß-actin

Figure 4A

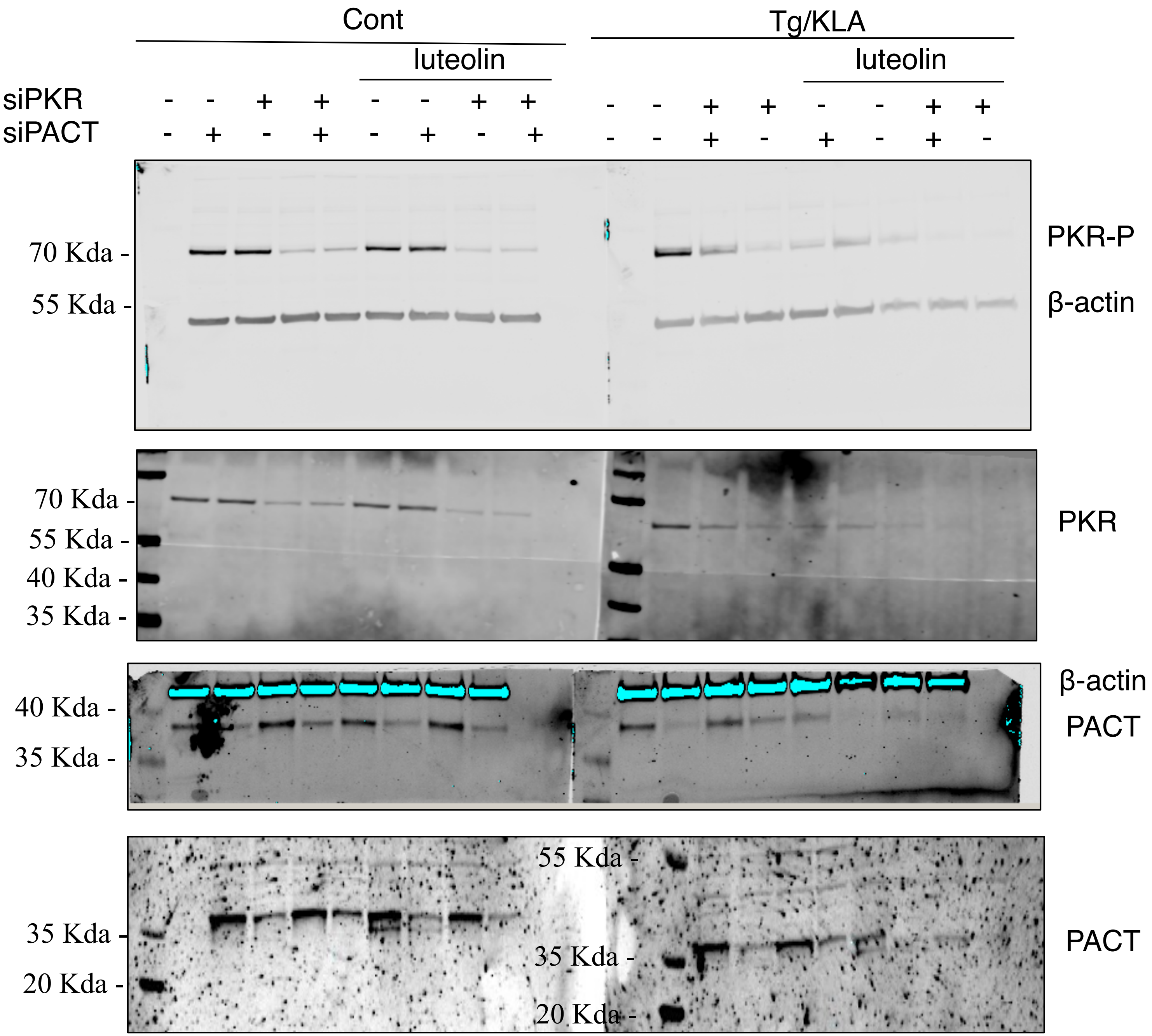
Cont
Tg/KLA
luteolin
luteolin
siPKR
siPACT
70 Kda -
55 Kda -
PKR-P
β-actin
70 Kda -
55 Kda -
40 Kda -
35 Kda -
PKR
40 Kda -
35 Kda -
β-actin
PACT
35 Kda -
20 Kda -
55 Kda -
35 Kda -
20 Kda -
PACT

Figure 8 A

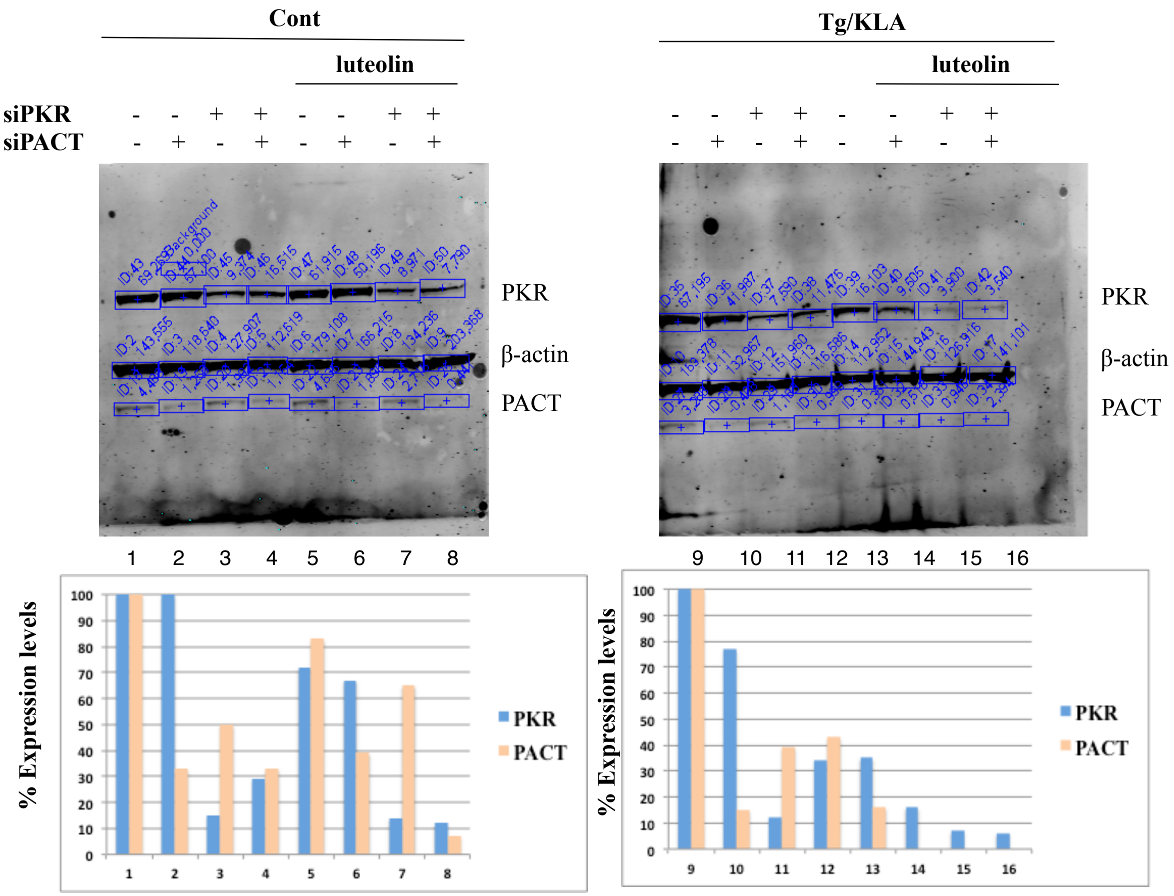
Cont
luteolin
Tg/KLA
luteolin
siPKR
siPACT
PKR
β-actin
PACT
1 2 3 4 5 6 7 8
9 10 11 12 13 14 15 16
% Expression levels
PKR
PACT
